# HIV envelope antigen valency on peptide nanofibers modulates antibody magnitude and binding breadth

**DOI:** 10.1038/s41598-021-93702-x

**Published:** 2021-07-14

**Authors:** Chelsea N. Fries, Jui-Lin Chen, Maria L. Dennis, Nicole L. Votaw, Joshua Eudailey, Brian E. Watts, Kelly M. Hainline, Derek W. Cain, Richard Barfield, Cliburn Chan, M. Anthony Moody, Barton F. Haynes, Kevin O. Saunders, Sallie R. Permar, Genevieve G. Fouda, Joel H. Collier

**Affiliations:** 1 Department of Biomedical Engineering, Duke University, 101 Science Dr., Campus, Box 90281, Durham, NC 27708 USA; 2 Department of Molecular Genetics and Microbiology, Duke University School of Medicine, Durham, NC 27710 USA; 3 Duke Human Vaccine Institute, Duke University School of Medicine, Durham, NC 27710 USA; 4 Department of Biostatistics and Bioinformatics, Duke University School of Medicine, Durham, NC 27710 USA; 5 Department of Pediatrics, Duke University Medical Center, Duke University School of Medicine, Box 103020, Durham, NC 27710 USA; 6 Department of Immunology, Duke University School of Medicine, Durham, NC 27710 USA; 7 Department of Surgery, Duke University School of Medicine, Durham, NC 27710 USA; 8 Department of Pediatrics, New York-Presbyterian/Weill Cornell Medicine, New York, NY 10065 USA

**Keywords:** Biomaterials, Biomaterials, Vaccines, HIV infections

## Abstract

A major challenge in developing an effective vaccine against HIV-1 is the genetic diversity of its viral envelope. Because of the broad range of sequences exhibited by HIV-1 strains, protective antibodies must be able to bind and neutralize a widely mutated viral envelope protein. No vaccine has yet been designed which induces broadly neutralizing or protective immune responses against HIV in humans. Nanomaterial-based vaccines have shown the ability to generate antibody and cellular immune responses of increased breadth and neutralization potency. Thus, we have developed supramolecular nanofiber-based immunogens bearing the HIV gp120 envelope glycoprotein. These immunogens generated antibody responses that had increased magnitude and binding breadth compared to soluble gp120. By varying gp120 density on nanofibers, we determined that increased antigen valency was associated with increased antibody magnitude and germinal center responses. This study presents a proof-of-concept for a nanofiber vaccine platform generating broad, high binding antibody responses against the HIV-1 envelope glycoprotein.

## Introduction

HIV-1/AIDS has been responsible for 30 million deaths since it became a global pandemic^1^. Despite decades of research, a vaccine against HIV-1 has remained elusive. A central challenge to developing a vaccine against HIV-1 is the genetic diversity of the virus, representing a moving target for vaccination. Across time, geographic regions, and even within infected individuals the antigens on the envelope of the HIV virus undergo significant evolution. For example, there is as much as 30% sequence diversity in HIV-1 envelope proteins across different strains of the virus^2^ and once infection is initiated, viruses rapidly mutate to evade immune recognition during the first few weeks of infection^3^. However, about half of HIV-infected individuals develop broadly neutralizing antibodies (bNAbs)^4^, which are capable of neutralizing diverse strains of HIV-1 via binding to specific conserved epitopes. Consequently, a major goal of HIV vaccine researchers is to develop a vaccine capable of inducing antibody responses which can inhibit HIV viruses of broad genetic diversity.

A major effort toward developing a protective HIV vaccine has been to engineer antigens and vaccine constructs with structural features to maximize generation of broad immune responses. In addition to engineering single antigens which steer antibodies toward neutralizing epitopes^5–7^, immunization regimens which combine antigens from multiple subclasses of HIV have been utilized to improve the breadth of cellular and humoral immune responses^8–10^. Activating low-affinity B cells early in the immune response may be a crucial factor in generating an efficacious HIV vaccine, as many B cells with low affinity to full HIV trimers are often the precursors for bNAb-producing B cell clones^7^. Antigens arrayed on nanomaterials or other surfaces are able to lower the threshold of B cell activation by clustering multiple B cell receptors, allowing for activation of B cell clones which would likely not be activated by soluble antigen^11^. The display of immunogens on nanoparticles has also been demonstrated to increase the breadth of antibody responses to HIV envelope trimers^12,13^. Nanomaterial-based displays of antigens have also showed benefits in increasing trafficking to germinal centers^14^, germinal center responses^15^, and B cell activation^16^. For HIV antigens arrayed on liposomes, antigen spacing was found to correlate directly with antigen titer, with titer maximized when antigens were spaced 15 nm apart^12^. The number of antigens displayed on nanoparticles also correlates to the magnitude of antibodies and germinal center responses that these vaccines elicit, as elegantly demonstrated in a two-component protein nanoparticle system^17^. Recently, antigen valency was also demonstrated to have direct impacts on the breadth of B cells which respond to multivalent immunogens by altering the affinity range of B cells which are activated after immunization^18^.

Although nanoparticle vaccines have demonstrated several benefits over immunization with soluble proteins, existing approaches have not yet been able to consistently generate broad antibody responses. Because of this, new technologies are needed to investigate possible strategies for generating immune responses against HIV antigens with high potency and breadth. Peptide nanofibers have demonstrated unique immunogenicity in contexts where very few, and likely low-affinity B cell clones, exist. These include tolerized epitopes such as those from autologous proteins such as TNF^19^ or IL-17^20^. Additionally, nanofiber vaccines can be administered mucosally^21,22^ and do not require additional adjuvants to raise high-titer antibody responses^23^, features which may be advantageous for a vaccine to be used in low-resource settings. For these reasons, we have constructed proof-of-concept nanofiber vaccines displaying the HIV envelope antigen gp120. Thus far, peptide nanofibers have largely been utilized to generate immune responses to specific peptide epitopes, but herein we describe their utility for generating immune responses against a folded glycoprotein antigen. We demonstrate that nanofiber-displayed antigens are associated with an increased magnitude and breadth of antibody responses compared to soluble gp120 antigens. These effects were associated with the density of antigens on nanofibers, where increased antigen density was associated with increased antibody titers and induction of T follicular helper (T_FH_) cells.

## Results

### Construction and antigenicity of gp120-nanofiber conjugates

**Figure 1:**
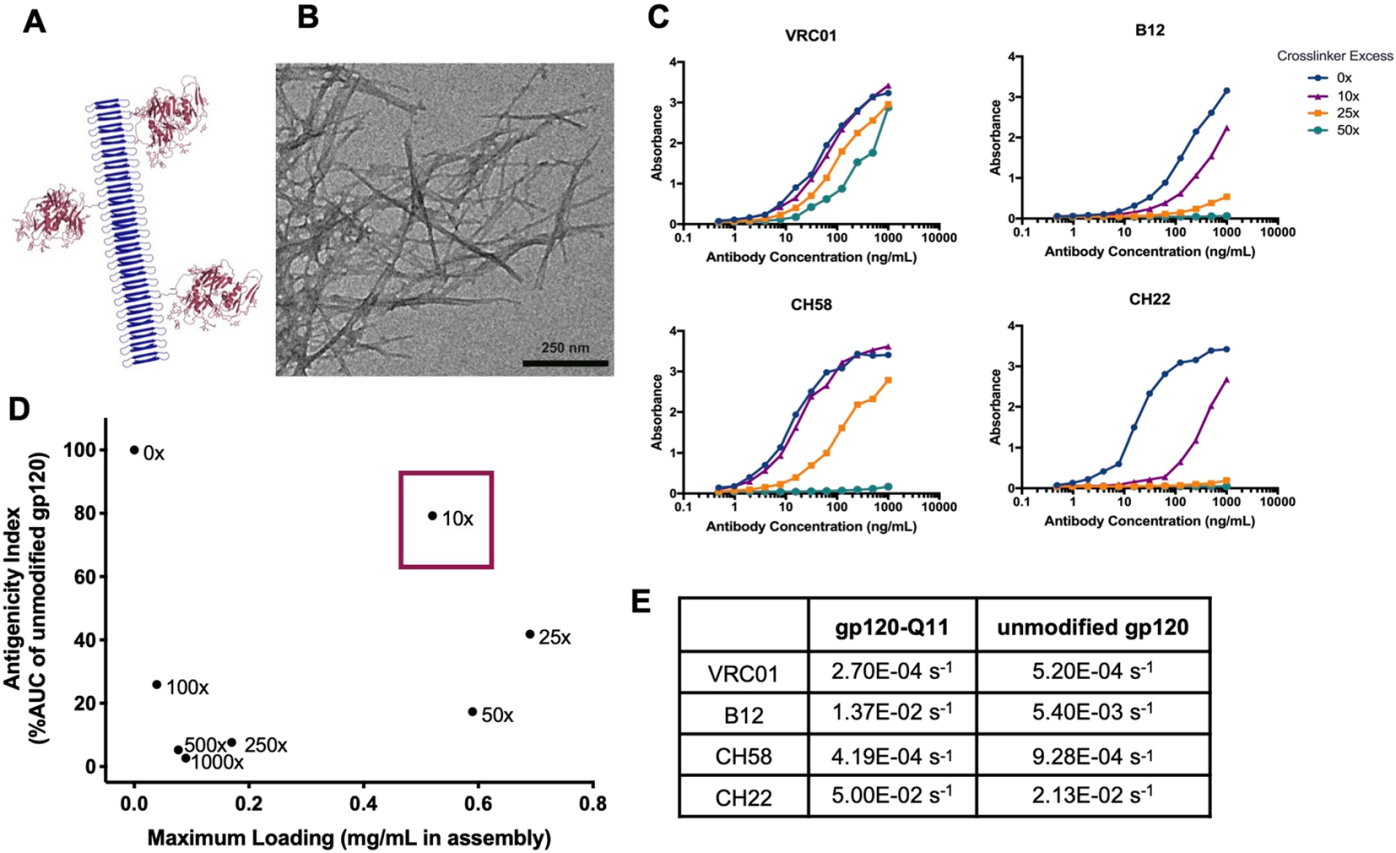
Nanostructure and antigenicity of nanofiber-gp120 conjugates. (**A**) Schematic representation of gp120 antigens (maroon) covalently linked to fibrillizing peptides (blue). (**B**) TEM image of gp120 nanofibers stained with uranyl acetate. (**C**) Binding of antibodies against VRC01, B12, CH58, and CH22 epitopes to gp120 nanofibers prepared with different molar ratios of crosslinking agent SMCC, measured by ELISA. (**D**) Representation of gp120 antigenicity and loading density on nanofibers with selected formulation (10×) boxed in red. Antigenicity index for each condition was calculated by adding the AUC of ELISA binding curves from VRC01, B12, CH58 and CH22, then dividing by the sum AUC of unmodified gp120. (**E**) Dissociation rate constants (k_off_) of antibodies to gp120 and gp120 nanofibers measured by Biolayer Interferometry.

Before investigating the immunogenic properties of gp120-nanofibers, conjugation chemistry was optimized to generate the constructs pictured in Fig. 1. The peptide nanofibers utilized in this study are based on the self-assembling peptide Q11 (all peptide sequences included in Table S1), which forms β-sheet fibrils in aqueous solution (Fig. 1A,B)^24^. To provide a reactive group adjacent to the self-assembling motif, the Q11 sequence was extended to include a N-terminal cysteine residue (C-Q11). To form gp120-nanofiber conjugates, gp120 from the transmitted/founder clade C strain 1086.C^25^ was modified with a heterobifunctional crosslinker (sulfo-SMCC), dialyzed to remove excess crosslinker, mixed with C-Q11 fibers to form covalent linkages, and nanofibers were pelleted by simple centrifugation to separate unconjugated antigens. A range of stoichiometries were tested for modifying gp120 with sulfo-SMCC and found to have a considerable effect on the yield and antigenicity of gp120 proteins that were linked to nanofibers after conjugation.

We quantified antigenicity of the nanofiber-conjugated gp120 by testing the ability of monoclonal antibodies specific to the gp120 component of the HIV envelope to bind to the vaccine constructs by ELISA. These antibodies had very low background binding to C-Q11 nanofibers alone (Fig. S1). Increasing molar ratios of sulfo-SMCC diminished binding of antibodies to gp120, with minimal perturbation of binding achieved at a tenfold excess of SMCC (Fig. 1C). The panel of antibodies were selected for binding to different domains of gp120; VRC01 and B12 react with the CD4 binding site that initiates fusion to target cells, CH58 binds to the second variable (V2) loop of gp120 which was targeted by antibodies associated with protection in the RV144 trial^26^, and CH22 binds the tip of the third variable (V3) loop of gp120, which is a major epitope on the HIV-envelope which was also associated with protection in the RV144 trial^27^. Of the antibodies tested, CH22 was the most sensitive to perturbation by crosslinker stoichiometry. This may be because the linear epitope of CH22 (RKRIHIGPGRAFYTT) includes a lysine residue, which was likely modified by SMCC to greater extents as crosslinker stoichiometry was increased. Interestingly, the epitope of CH58 contains multiple lysines (KKKVHALFYKLDIV) but was not as strongly affected by crosslinking as the CH22 epitope, potentially because it is less reactive to SMCC modification, or because CH58 binding may be less dependent on contacts with lysine residues. High amounts of crosslinker also decreased the amount of gp120 that was conjugated to nanofibers, likely by crosslinking multiple cysteine residues on fibers with a single antigen, limiting the availability of cysteines for conjugation to other antigens. The interplay of gp120 valency and antigenicity is displayed in Fig. 1D, indicating that formulations with a tenfold excess of sulfo-SMCC maximized preservation of gp120 epitopes and allowed for the highest concentration of gp120 antigens displayed on nanofibers. By selecting formulations maximizing both antigenicity and valency, this increased the likelihood of capitalizing on the immunogenic benefits of multivalent antigen display that are described above. To overcome potential conformational impacts of plate-based ELISA measures of antigenicity, the binding of gp120-nanofibers was also quantified by a solution-phase measurement using biolayer interferometry (BLI). The BLI binding analysis showed similar dissociation constants (k_off_) for unmodified gp120 and gp120-nanofibers (Fig. 1E). The association rate (k_on_) of antibodies to gp120-nanofibers was slower than association to gp120 alone (Fig. S2), likely because nanofibers sterically hinder antibody binding. Thus, in the remaining investigations, a tenfold excess of sulfo-SMCC was used to generate gp120-nanofiber immunogens as they retained binding to linear epitopes of gp120 and maximized its valency on nanofibers.

### Antibodies raised by gp120-nanofiber conjugates have higher magnitude and binding breadth than those generated by soluble gp120

**Figure 2:**
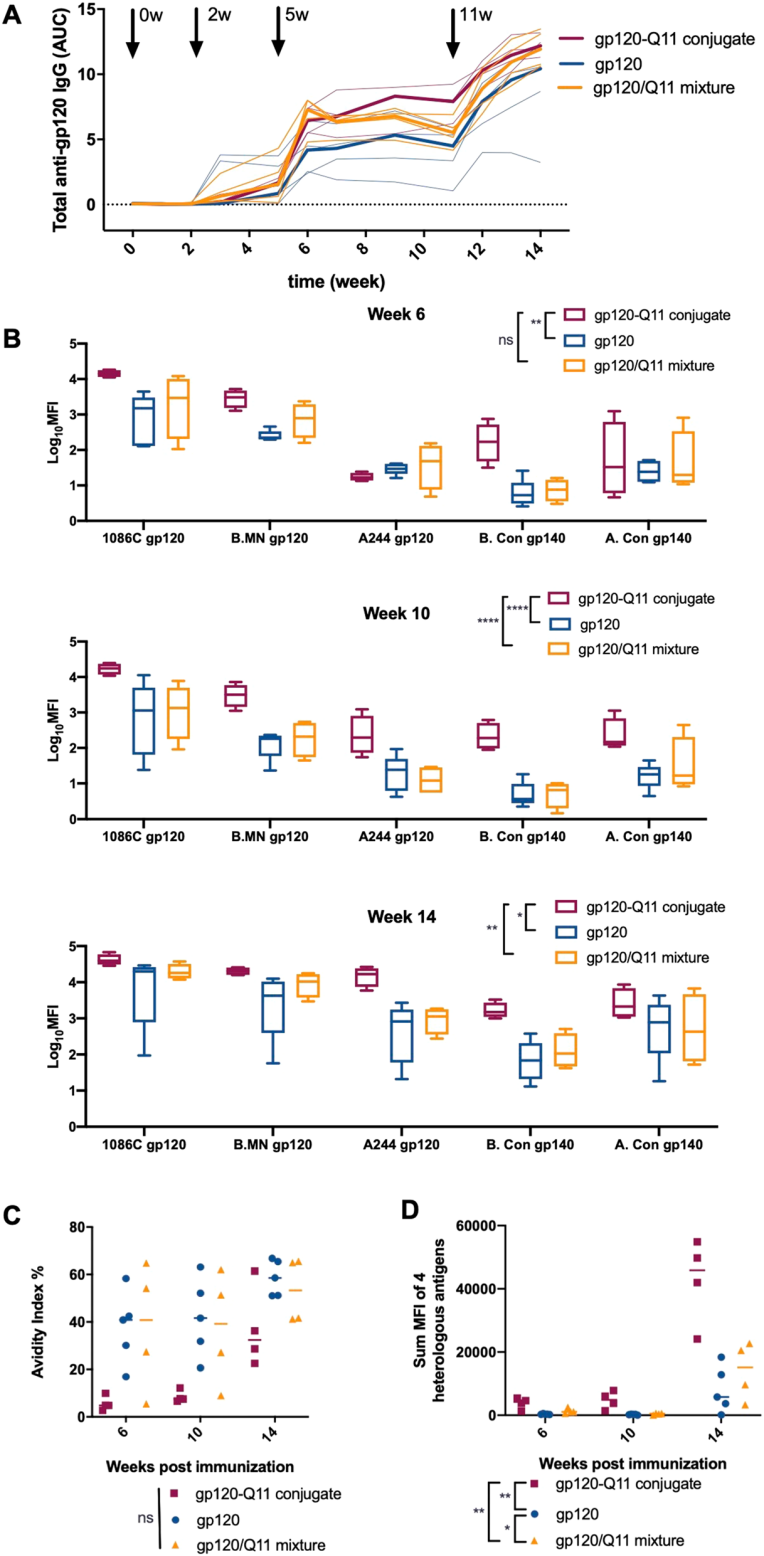
Nanofiber conjugation increases antibody binding breadth. (**A**) Magnitude of serum antibody responses to gp120 from mice immunized with gp120 or gp120 nanofibers at times indicated by arrows as measured by ELISA. Bold line shows mean titer and thin lines show individual responses. Mice received 4 total immunizations of 50 μg gp120 each. Responses were compared using a linear mixed effects model and no significant difference was observed. (**B**) Binding of serum antibodies to a panel of heterologous antigens at 6, 10, and 14 weeks post immunization. Groups were compared using GEE (see methods for more detail). (**C**) Avidity index of serum antibodies to gp120 measured by antibody binding under denaturing conditions. (**D**) Time progression of heterologous antigen binding displayed by summing the responses shown in (**B**). Groups were compared using a repeated measures non-parametric test with differences between treatment groups denoted on graph legends. *p < 0.05, **p < 0.01, ****p < 0.0001. (n = 4 animals/group for gp120-Q11 and gp120/Q11, n = 5 animals/group for gp120).

To study the immunogenicity impacts of covalently linking gp120 to nanofibers,C57BL/6 mice were immunized with either soluble gp120, gp120 covalently linked to Q11 nanofibers as described above (gp120-Q11), or gp120 physically mixed with Q11 fibers with no chemical modification (gp120/Q11). When used as an immunogen, 4 doses of gp120 covalently linked to Q11 or simply mixed with Q11 (gp120-Q11 and gp120/Q11, respectively) induced a modest but non-significant increase in the magnitude of anti-gp120 IgG responses compared to soluble gp120 (immunizations given at 0, 3, 5, and 11 weeks) (Fig. 2A). The most striking feature of the antibodies induced by gp120-Q11 nanofibers, however, was their increased binding to a panel of heterologous HIV gp120 antigens compared to that of soluble gp120 (Fig. 2B, *p* values between 0.01 and 0.00005 depending on time point). In order to probe the binding breadth of the antibodies elicited by gp120 vaccines, we used a gp120 binding antibody multiplex assay (BAMA), a bead-based antibody binding assay that allows measurement of binding to multiple antigens in a single assay^28–30^. We selected the strains of HIV envelope antigens to cover the dominant clades that circulate in different geographic areas, clade B (North, Central, and South Americas), clade A (East Africa, East Europe, and Central Asia), and CRF_AE (East and Southeast Asia)^31^. The antigens include MN.B gp120 (clade B), B. Con gp140 (clade B), A. Con gp140 (clade A), and A244 gp120 (CRE_AE). Binding to 1086.C gp120 measured by ELISA and BAMA techniques were correlated at all time points measured in this study (Fig. S3), and we observed similar trends in the binding of mAbs to heterologous antigens using BAMA and ELISA (Fig. S4). Additionally, we utilized BAMA for multiplexed antigen binding because of its sensitivity over a wide dynamic range (Fig. S5)^32–34^.

Though covalent conjugation of gp120 to Q11 nanofibers did not significantly improve antibody magnitude in comparison to gp120 mixed with Q11 fibers (Fig. 2A), it had a significant impact on the heterologous binding profile of elicited antibodies (Fig. 2B), a critical feature for potential HIV vaccine candidates. Early in the immune response, antibodies elicited by gp120-Q11 nanofibers had a slightly lower avidity index than those induced by soluble gp120 and gp120/Q11 (Fig. 2C). However, after multiple boosts, the antibodies induced by gp120-nanofibers increased in avidity though they were still lower than those induced by soluble gp120 or gp120/Q11 (Fig. 2C). Overall, we did not observe a statistically significant association with response and avidity (p-value of 0.08). The binding breadth of these antibodies increased alongside their avidity, with early increases observed for mice immunized with gp120-Q11 continuing to surpass the breadth of antibodies induced by soluble gp120 or gp120/Q11 (Fig. 2B,C,D). These findings suggest that covalent linkage of gp120 to Q11 nanofibers was critical in generating antibodies with a broad heterologous binding profile. Because heterologous binding breadth was the feature of antibody responses we sought to maximize, we focused subsequent investigations on chemically conjugated gp120-Q11 nanofibers.

**Figure 3:**
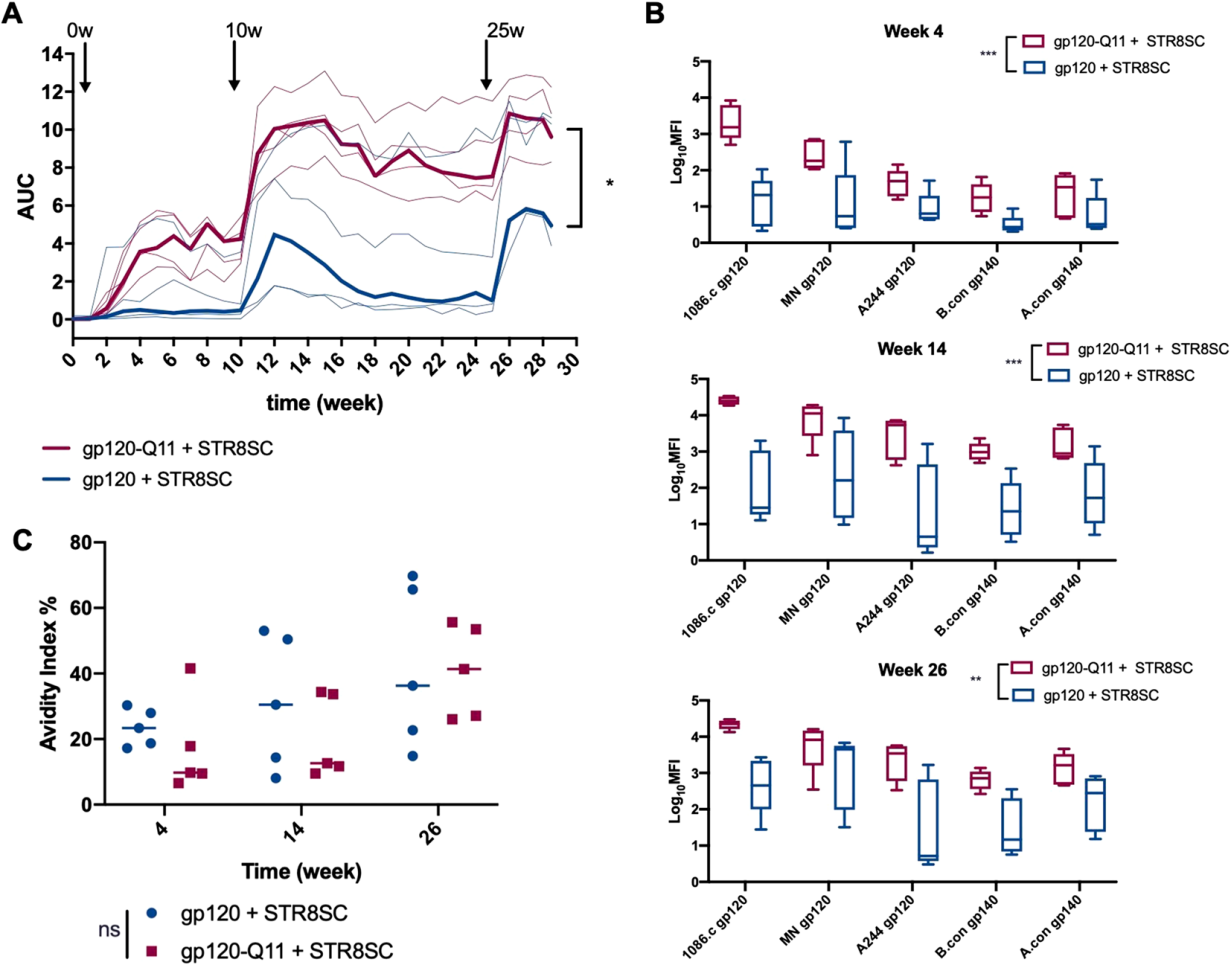
Multivalent display of proteins increases titers and heterologous binding when formulated with adjuvant. (**A**) Magnitude of serum antibody responses to gp120 from mice immunized with gp120 or gp120 nanofibers adjuvanted with STR8SC at times indicated by arrows as measured by ELISA. Bold line shows mean titer and thin lines show individual responses. Mice received 3 total immunizations of 15 μg gp120 each. Compared using a linear mixed effects model, *p < 0.05. (**B**) Binding of serum antibodies to a panel of heterologous antigens at 4, 14, and 26 weeks post immunization. Groups were compared using GEE (see methods for more detail). **p < 0.01, ***p < 0.001. (**C**) Avidity index of serum antibodies to gp120 measured by binding of antibodies under denaturing conditions. Groups were compared using a repeated measures non-parametric test and no significant difference was found.

Because conjugating gp120 to Q11 nanofibers showed promising results in our preliminary animal studies, we tested these materials incombination with an adjuvant, since adjuvants are routinely used to improve immune responses in clinical settings. While we observed a modest increase in antibody titer and early binding breadth when Q11 nanofibers were mixed with gp120, gp120-Q11 conjugates increased antibody binding breadth to a greater extent, so we excluded physically mixed gp120/Q11 in this study (Fig. 2). gp120-Q11 improved antibody binding breadth compared to gp120 when STR8SC adjuvant was incorporated with immunizations (Fig. 3). STR8SC is an oil-in water emulsion containing TLR9 and TLR7/8 agonists that is capable of eliciting potent antibody responses to HIV Envelope vaccines in non-human primates and in C57BL/6 mice^35,36^ and has been used with 1086.C gp120 in other studies^37–39^. Using a less aggressive boosting schedule (3 doses given at 0, 10, and 24 weeks) and lower antigen dose than in initial studies, gp120-Q11 induced an increase in IgG binding magnitude over the course of the immunization regimen (Fig. 3A, *p* value = 0.027). The antibody response induced by adjuvanted gp120-Q11 also showed statistically significantly broader binding to heterologous antigens than those induced by adjuvanted soluble gp120 (Fig. 3B, *p* values between 0.002 and 0.001 depending on time point). At a lower antigen dose, IgG responses elicited by adjuvanted gp120 and gp120-Q11 showed no statistically significant difference in binding avidity, even with fewer boosting immunizations (Fig. 3C, *p* =0.457). While the exact mechanism by which nanofiber-based vaccination increases antibody binding breadth is unclear, the increased binding breadth observed by gp120-Q11 elicited antibodies could be due to a shift toward more conserved epitope specificities, a more diverse antibody repertoire directed towards a variety of sites on gp120, or to increased antibody affinity maturation. Inflammatory responses towards Q11 may play a modest role in antibody production, but we have previously measured lower levels of inflammatory cytokines after Q11 immunization compared to other adjuvants such as Alum or SAS while recruiting APCs and initiating B and T cell responses^40^ and modest levels of anti-Q11 antibodies were measured in this system and others (Fig. S6). Taken together, these results indicated that nanofiber display of gp120 is associated with increased IgG antibody magnitude and breadth without compromising binding avidity.

### High antigen valency drives the breadth and magnitude of vaccine-elicited IgG and germinal center responses

**Figure 4:**
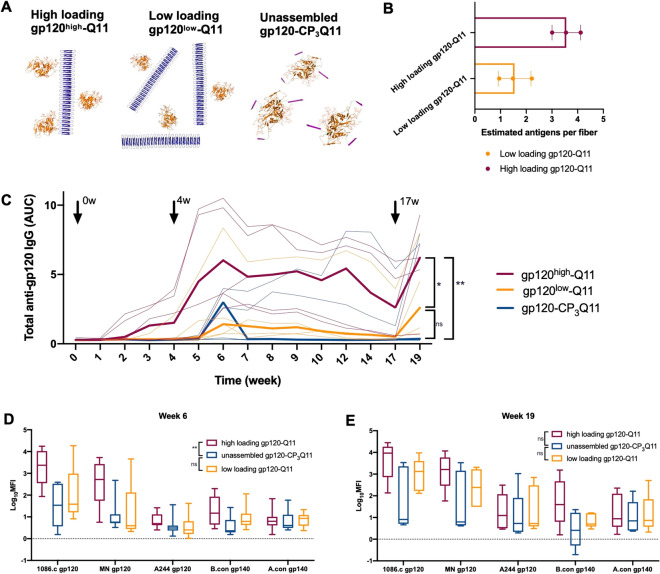
High gp120 antigen density on nanofibers drives increased antibody titer and binding breadth. (**A**) Schematic representation of gp120 nanofibers and unassembled antigens modified with non-assembling peptides. (**B**) Number of gp120 antigens on gp120^high^-Q11 and gp120^low^-Q11 for 3 immunizations estimated by gp120 concentration and average fiber length. (**C**) Magnitude of serum antibody responses to gp120 from mice immunized with indicated formulations at times indicated by arrows as measured by ELISA. Bold line shows mean titer and thin lines show individual responses. Compared using a linear mixed effects model, *p < 0.05,**p < 0.01. (**D**) Binding of serum antibodies to a panel of heterologous antigens at 6-weeks post primary immunization. (**E**) Binding of serum antibodies to heterologous antigens from 19-weeks post primary immunization as in D. Groups were compared using GEE (see methods for more detail). **p < 0.01.

Although gp120-Q11 offered significantly improved IgG responses compared to gp120, it was unclear what features of Q11 nanofiber-based vaccination afforded these advantages. Although multiple factors such as trafficking, epitope spacing, and antigen density may be at play^41^, we specifically investigated the impact of antigen density and valency on the nanofibers on the IgG response profile. To test the impact of multivalency on gp120-Q11 vaccines, we synthesized Q11 nanofibers with high and low densities of gp120, and modified gp120 with a non-assembling peptide to serve as a control (Fig. 4A). After studying the antibody responses displayed in Fig. 2, we hypothesized that some level of non-specific adsorption of gp120 to Q11 nanofibers was occurring and inducing a modest level of adjuvanting to gp120/Q11 mixtures, possibly rendering this formulation with some degree of multivalency. We have also noted such non-specific adsorption for smaller proteins mixed with Q11^42^, and expect that the increased size and surface area of gp120 may have contributed to this effect. Additionally, we sought to include a control which accounted for the modification of gp120 epitopes by SMCC crosslinkers, which could have influenced the epitope specificity and resultant binding breadth for gp120-Q11 induced antibodies. Thus, to incorporate this control, we modified gp120 with a non-assembling peptide (gp120-CP_3_Q11). High and low loading constructs were made by altering the stoichiometry of SMCC-modified gp120 and C-Q11 peptides incorporated in nanofibers during synthesis and the final concentration of gp120 on nanofibers was measured by SDS-PAGE (Fig. S7). Based on the concentration of gp120 in each formulation and the length of resultant gp120-Q11 nanofibers (Fig. S8), high loading gp120-Q11 (gp120^high^-Q11) nanofibers were estimated to have an average of 3–4 antigens per fiber, and low loading gp120-Q11 (gp120^low^-Q11) was estimated to have an average of 1–2 antigens per fiber (Fig. 4B). Non-assembling controls were synthesized by conjugating a non-assembling variant of the Q11 sequence termed CP_3_Q11, wherein phenylalanine residues in the Q11 sequence were mutated to proline, disrupting its self-assembling properties^43^. The estimated antigens per fiber for each study is listed in Table S2.

These three constructs, unassembled gp120 (gp120-CP_3_Q11), low-loading gp120 nanofibers (gp120^low^-Q11), and high-loading gp120 nanofibers (gp120^high^-Q11), were administered to mice with equal gp120 content. They elicited distinctly different antibody responses. High-loading gp120-Q11 induced a striking increase in gp120-specific IgG response magnitude relative to both low-loading gp120-Q11 and non-assembling gp120-CP_3_Q11 (Fig. 4C, high loading vs. unassembled *p*=0.003, high loading vs. low loading *p=*0.041 after multiple testing correction), while antibody levels induced by low-loading gp120-Q11 were similar to those elicited by non-assembling gp120-CP_3_Q11 vaccines (*p*=0.231). IgG responses generated by high-loading gp120-Q11 developed binding breadth earlier in the immunization regimen than non-assembling gp120-CP_3_Q11 vaccines (*p*=0.003 for high loading vs. unassembled), though statistically significant differences in antibody binding breadth were not apparent after 3 immunizations (Fig. 4D,E). These results suggest that vaccine-elicited IgG magnitude and breadth were associated with antigen valency.

**Figure 5:**
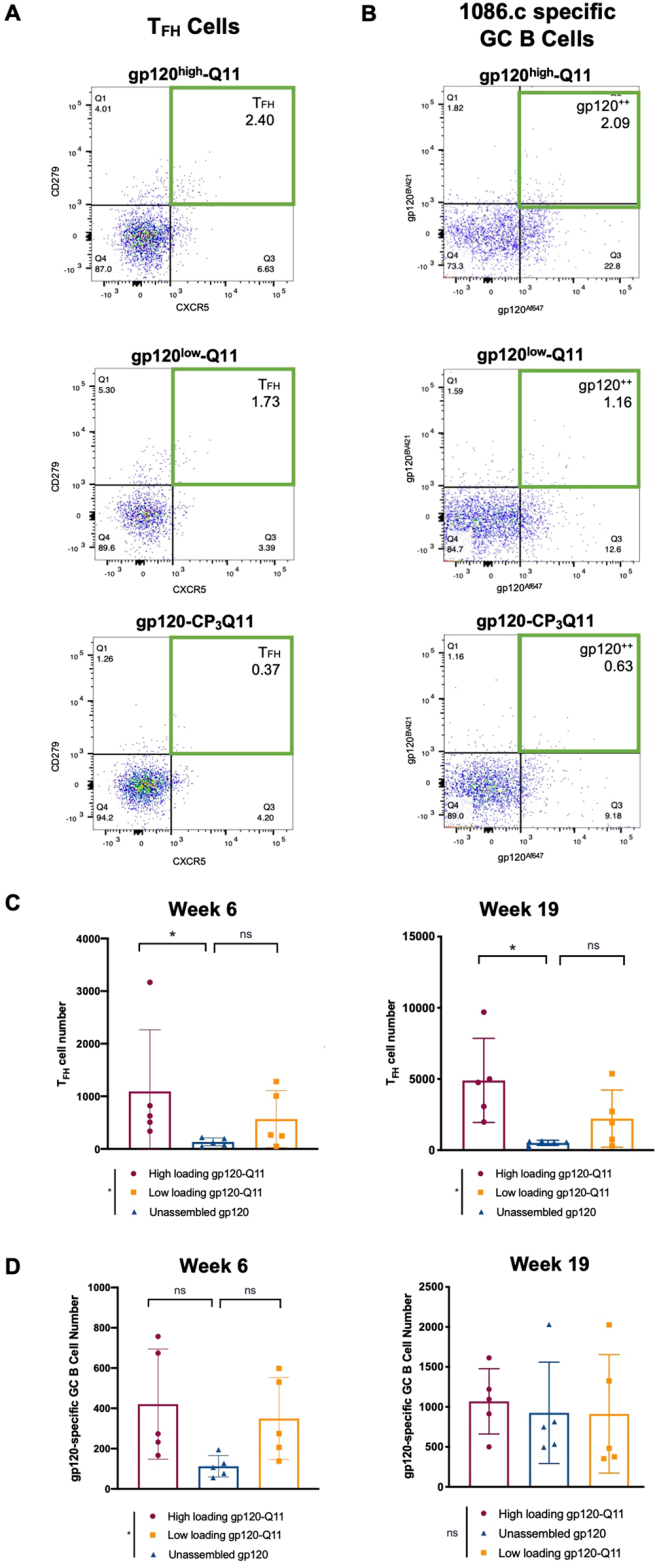
Antigen density on nanofibers modulates T_FH_ and GC B cell numbers. (**A**) Representative dot plots of CD3^+^ CD4^+^ CD44^hi^ CD62L^Lo^ CD25^-^ CD279^hi^ CXCR5^hi^ T follicular helper (T_FH_) cells detected by flow cytometry at 6-weeks post priming immunization (gating strategy shown in Fig. S11). (**B**) Representative dot plots detecting gp120-specific CD138^-^ B220^+^ GL7^hi^ germinal center B cells detected by flow cytometry at 6-weeks post priming immunization (gating strategy shown in Fig. S12). (**C**) T_FH_ cells from 6- and 19-weeks post primary immunization. In C and D, ranks were compared using the Kruskal–Wallis test to determine if there were significant differences among the groups, followed by post-hoc pairwise comparisons to test for differences between specific groups. Differences due to treatment group are shown in figure legends and differences between specific groups are shown above the data. (**D**) gp120-specific germinal center B cells at 6- and 19-weeks post primary immunization. The Kruskal–Wallis test indicated significant differences existed between treatment groups (*p* = 0.04, indicated next to the figure legends), but Holm post-hoc pairwise comparisons were not statistically significant (*p < 0.05, indicated above the data). n = 5 animals/group.

Varying antigen density in gp120-Q11 vaccines also led to changes in the number of T follicular helper (T_FH_) cells and gp120-specific germinal center B cells in the lymph nodes of immunized mice (Figs. 5, S9, S10, S11, S12). Statistically significant differences in T_FH_ cell numbers in the lymph nodes of immunized mice were observed after the first and second boosting immunizations (Fig. 5A,C, p=0.04 at week 6, *p*=0.03 at week 19) and differences in GC B cell numbers elicited by each immunization were observed early in the immunization regimen (Fig. 5B,D, p=0.04 at week 6, non-significant pairwise comparisons). High-loading gp120-Q11 nanofibers elicited statistically significantly higher numbers of T_FH_ cells than unassembled gp120-CP_3_Q11 (*p*=0.048), whereas low-loading gp120-Q11 nanofibers did not (*p*=0.286 after multiple testing correction) (Fig. 5C). Similarly, immunization with high-loading gp120-Q11 resulted in a higher percentage of CD4^+^ T cells exhibiting a T_FH_ phenotype (Fig. S9). After one boosting immunization, both high-loading gp120-Q11 and low-loading gp120-Q11 induced slightly higher numbers of gp120-specific germinal center B-cells than gp120-CP_3_Q11, though individual post-hoc comparisons between immunization groups did not yield statistically significance (*p*=0.08 for high loading vs. unassembled and low-loading vs. unassembled), likely due to low sample sizes (Fig. 5B,D). This potential accelerated initiation of germinal center B cell responses may be associated with early development of antibody binding breadth, which was observed for antibodies analyzed by BAMA (Fig. 4D) but would require more highly powered experiments to ascertain. Interestingly, the improvement in IgG magnitude invoked by high loading gp120-Q11 (Fig. 4C) was not solely tied to GC B cell responses, as evidenced by the similar levels of GC B cell numbers elicited by high- and low-loading gp120-Q11, and may be tied to other immunological phenomena such as plasmablast generation^18^. This data suggested the boosted germinal center responses often observed for nanomaterial vaccines may be tied to material-related factors other than antigen spacing, such as complement binding or altered trafficking from injection sites^14^. Additionally, the increase in GC B cell and T_FH_ cell numbers early in the immune response points towards differences in the development of activated B cells, which can lead to changes in the B cell repertoire and the diversity of elicited antibodies^44^.

**Figure 6:**
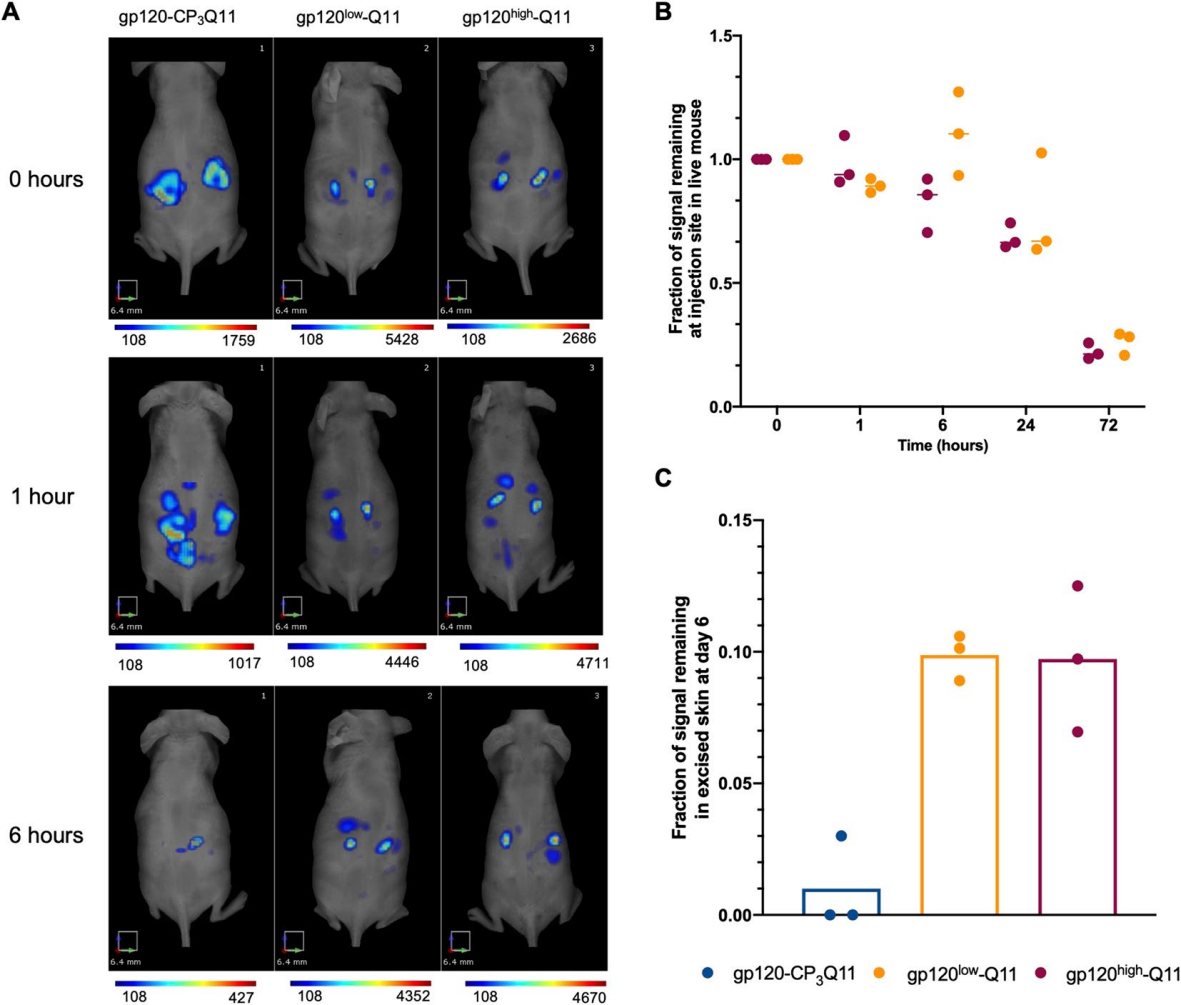
Nanofiber immunizations persist at injection sites independent of antigen loading density. (**A**) Representative FMT images of mice injected with fluorescently labeled gp120 or gp120 nanofibers at 0- and 1- and 6-h post injection. (**B**) Fluorescence signal at injection site for gp120^high^-Q11 and gp120^low^-Q11 immunizations for up to 72 h post injection. (n = 3 mice/group, compared using a repeated measures non-parametric test, no significant difference between groups). (**C**) Remaining fluorescent signal in skin sections at sacrifice, 6 days post-immunization. (n = 3 mice/group, compared by Kruskal–Wallis, *p* = 0.065).

Recent reports have noted the profound influence of multivalency on trafficking glycosylated antigens to germinal centers^14^. To determine if trafficking also played a role in the differential antibody magnitude and breadth observed for high-and low-loading gp120-Q11, we fluorescently labeled gp120-CP_3_Q11, gp120^low^-Q11, and gp120^high^-Q11 and monitored their drainage after subcutaneous injection in mice (Fig. 6). We noted rapid diffusion of gp120-CP_3_Q11 away from the injection site, which can be visualized by the more disperse localization of fluorescent material within 1 h after injection (Fig. 6A) and because of this rapid diffusion, an injection site depot could no longer be clearly identified at the skin surface as soon as 6 h post-injection (Figs. 6A, S13, S14). In contrast, both high- and low-loading gp120-Q11 remained at a clearly visualized injection site (Fig. 6A, S13) and drained over the course of 3 days (Fig. 6B). At 6 days post-injection, fluorescently labeled gp120-Q11 was still present at the injection site and could be visualized in the isolated skin of mice, whereas the skin of mice injected with gp120-CP_3_Q11 showed very low or undetectable signal (Figs. 6C, S15). Differences in the fluorescence signal of excised skin were compared using a Kruskal–Wallis test but did not reach significance, likely due to the low sample size (*p* = 0.07 for difference by treatment group). However, if nanofiber-treated mice are pooled into a single group, values were statistically significantly different (*p* = 0.03). These results suggest that the sustained availability of antigens displayed on nanofibers at injection sites may play a role in their efficacy compared to soluble proteins, potentially by extending the time course over which antigen can be delivered to downstream sites, particularly lymph nodes and germinal centers within them^45^. Although we did not directly measure the modes of antigen transport in this study, antigens may be transported to lymph nodes via free drainage through the lymphatics or by cell-mediated transport, as measured in other nanomaterial injections^46–48^.

## Discussion

The clade C HIV envelope glycoprotein (1086.C gp120) used in this study has been shown to induce strong antibody responses in mice when administered with Alum or AS01B adjuvant^49^ and in non-human primates when adjuvanted with ﻿3M-052-SE or STS/R848^50^. In this study, we demonstrate that conjugation of 1086.C gp120 antigens to Q11 nanofibers is associated with improved immune responses toward this antigen by increasing the magnitude of antibody responses in adjuvanted formulations, and by improving the heterologous binding breadth of antibodies in both adjuvanted and unadjuvanted formulations. Our findings are complementary to recent studies utilizing liposomes and protein-based nanoparticle vaccines wherein antigen valency is correlated with increased antibody titer^12,17^ and increased B cell activation^12^. Additionally, recent work has shown that multivalent antigen displays have an increased ability to activate low-affinity B cells within germinal centers compared to monomeric antigen and will likely be useful for activating low-affinity bNab precursors^18^. The observation that multimerization of gp120 by Q11 conjugation led to the early development of antibody binding to heterologous HIV envelope antigens suggested that nanofiber-based gp120 immunogens may increase the clonal diversity of activated B cells and drive the affinity maturation of antibodies, which are essential pathways for the emergence of HIV neutralization in humans and non-human primates^51^.

Nanofiber-based immunogens have been used extensively to elicit antibody responses to short, linear peptide epitopes^19,52,53^, but here we demonstrate their utility in raising IgG responses of high magnitude and breadth against a folded protein with multiple conformational epitopes. Capitalizing on the ability of highly multivalent displays to activate rare B cells with low-affinity precursors^19^, we found that peptide nanofiber-based vaccines can generate antibodies which bind genetically diverse antigens. After observing statistically significant changes in antibody binding breadth when comparing gp120-Q11 to gp120, we considered the possibility that the non-specific modification of lysine residues with SMCC might be responsible for the shifted antibody repertoire observed. However, the minimal gp120-specific IgG breadth observed in mice immunized with the non-assembling control gp120-CP_3_Q11 indicated that this was not the case. Previously, we have also not observed antibodies binding to the self-assembling peptide Q11^24^ and do not expect that anti-Q11 antibodies would be responsible for altering antibody responses to nanofiber-based immunogens. Additionally, antibodies reactive to Q11 were measured in pre-immune and endpoint serum and found to be 200-fold lower than responses to gp120 after 3 immunizations (Fig. S6). Even when gp120 epitopes were altered by SMCC modification, monovalent gp120 immunogens were outperformed by multimerized gp120-Q11.

Multiple factors may have been at play in causing the difference in IgG response magnitude and breadth between high- and low-loading gp120-Q11 (Fig. 4). Previously it has been shown that nanomaterial size and antigen valency influences trafficking to germinal centers ^14^, and although there were minor differences in the length distributions of high- and low-loading gp120-Q11 nanofibers, no differences were observed in their trafficking properties at a macroscopic level (Fig. 6). Because similar kinetics of injection site drainage were observed for high- and low-loading gp120-Q11 nanofibers, we expect that their differential ability to initiate antibody responses of high magnitude and binding breadth was due to their interactions with individual B cells and other APCs rather than any differential trafficking properties. Other studies have systematically evaluated the impact of antigen valency on multiple features of humoral responses which may impact responses to gp120-Q11 immunogens^12,18^. Specifically, increased antigen valency is associated with increased activation of B cells^12^ and can also stimulate survival of low-affinity B cells within germinal centers and promote plasmablast generation in comparison to monovalent antigens^18^. One component of the humoral response which seems to be critically influenced by antigen valency is the induction of T_FH_ cell responses within germinal centers. We measured increased number of T_FH_ cells produced by high-density gp120-Q11, suggesting that future generations of these materials may have the potential increase T cell help for rare bNAb precursor B cells to generate broadly neutralizing antibodies, as T_FH_ responses are critical to promoting bNAb development^54^. In this proof-of-concept study, we did not directly measure HIV-1-neutralizing antibodies (Nabs) because of the gp120 antigen used and the limited ability of mice to generate NAbs relative to higher order species^55,56^. Nevertheless, the broad antibody binding induced by nanofiber immunogens warrants their development and investigation in contexts where NAb induction is more likely. To this end, future generations of nanofiber-based immunogens could be improved by incorporating native-like antigens, such as SOSIP trimers or engineered minimal antigens, such as eODGT8^57^, using site-specific conjugation chemistry to maximize their potential to generate bNAbs^58,59^. Further, the degree of multivalency achieved in this study was relatively modest and non-specific modification of lysine residues may not be an ideal approach for maximizing antigenicity in other vaccine applications. In principle, nanofibers with greater numbers of antigen copies may be achievable by further optimizing the conjugation chemistry or by developing alternative approaches for integrating the antigens into the nanofibers.

In addition to the strengthened immune responses promoted by gp120-Q11 immunogens, these materials may also lend themselves to implementation in limited-resource settings. The improvement of the magnitude and breadth of the antibody response to gp120-Q11 compared to gp120 after only two immunizations (Fig. 3), suggests that multivalent approaches such as the one reported here may have value in facilitating dose sparing and improving compliance. Fewer required boosts could minimize the overall cost of vaccine regimens and diminish the necessity for patients to adhere to prolonged boosting schedules, which may be particularly important in low-resource settings. In addition to these advantages, nanofiber-based vaccines have previously shown excellent thermal stability and may have reduced requirements for cold-chain distribution compared to existing vaccines^60^.

Herein, we describe the first use of peptide-nanofibers to display a folded glycoprotein antigen with conformational epitopes for the elicitation of immune responses against the HIV-1 envelope glycoprotein gp120. By minimizing the loss of epitope structures and maximizing antigen valency, we utilized these materials to generate antibodies with broad binding profiles to HIV envelope antigens not included in immunizations. Based on observed antibody titers, binding levels, and germinal center responses, we expect that multivalent nanomaterials will have a unique impact in the context of HIV and other infectious pathogens that display antigens of high genetic variability. Of particular current relevance, this finding may be translatable to other viruses such as influenza or coronaviruses.

## Methods

### Peptide synthesis and purification

The names, sources, and catalog numbers of key reagents are listed in the Supplemental Information. Peptides were synthesized via microwave-assisted solid phase synthesis using Fmoc-protected amino acids on a Liberty Blue Peptide Synthesizer (CEM). After synthesis, peptides were cleaved from resin using a cocktail of 95% trifluoroacetic acid, 2.5% water, and 2.5% triisopropyl silane and precipitated in diethyl ether. Desired sequences were then separated from off-target products using reverse-phase HPLC on a Waters XBridge C12 column using water-acetonitrile gradients. Peptide mass was confirmed using MALDI-ToF mass spectrometry. Purified products were lyophilized and stored at − 20 °C.

### Synthesis of gp120-nanofiber conjugates

To form C-Q11 peptide nanofibers, C-Q11 and Q11 were dissolved in water at 0.4 mM and 7.6 mM, respectively, and incubated at 4 °C overnight. The following day, water and PBS without calcium and magnesium (Corning 46-013-CM) were added to bring the solution to a 2mM peptide concentration in 1x PBS, and the nanofiber solution was stored at RT for 3 hours before use in conjugate syntheses. To produce gp120-Q11 conjugates, gp120 was first modified with a 10-fold molar excess of the crosslinker 4-(*N*-maleimidomethyl)cyclohexane-1-carboxylic acid 3-sulfo-*N*-hydroxysuccinimide ester (sulfo-SMCC, Thermo Fisher) dissolved in 10% DMSO, 90% PBS at pH 7. After rotating gp120 and SMCC at 4 °C for 2 hours on a rotary shaker, solutions were dialyzed in PBS at 4 °C for 3 hours to remove unreacted sulfo-SMCC. After dialysis, sulfo-SMCC-modified gp120 was mixed with C-Q11 nanofibers and rotated overnight at 4 °C. The following day, nanofibers were centrifuged at 9000×*g* for 7 min to form a pellet that physically resembled a hydrogel. The supernatant was removed, stored, and replaced with fresh PBS. Centrifugation and resuspension were repeated three times before use of the final product. To quantify the amount of gp120 on nanofibers, nanofiber products and gp120 standards of known concentration were analyzed by Coomassie-stained SDS-PAGE. Gels were imaged on a BioRad gel imager and images were analyzed by Image J. For each synthesis, standard bands were used to create a calibration curve for gp120 concentration and used to quantify the concentration of gp120 in nanofibers.

### TEM imaging and image analysis of gp120-nanofiber conjugates

Nanofiber conjugates were prepared as described above and diluted tenfold in PBS before spotting onto formvar-coated copper TEM grids. After incubating, grids were rinsed with several drops of DI water and then stained with 1% uranyl acetate. After staining, excess solution was wicked off of grids using a tissue. Imaging was performed on a FEI Tecnai TEM microscope at 160 kV and 10,000–100,000× magnification. Lengths of individual nanofibers were measured using ImageJ software. To estimate the number of gp120 antigens on high- and low-loading gp120-Q11 nanofibers, the relative concentration of gp120 (6.52 mM for high-loading, 1.06 mM for low-loading) to CQ11 and Q11 (2mM) was used to determine the average number of peptides between antigens. The spacing was then determined by converting peptide numbers to lengths based on the crystal structure of a similar β-sheet fiber (PDB ID 2nnt).

### ELISA analysis of gp120 structure

Antibody names, sources, and catalog numbers for ELISA and other immunoassays are listed in the Supplemental Information. Conjugated Q11 nanofiber and unconjugated 1086.C gp120 (9 mg/mL, 135 ng/well) were coated on 384-well polystyrene high-binding plates (Corning) in coating solution (Seracare, Milford, MA) at 4°C overnight. The plates were blocked with superblock solution (4% whey, 15% goat serum, 0.5% Tween 20 in 1xPBS) for 1 hour. Two-fold serial dilution starting at 1 μg/ml of house-made HIV-1 monoclonal antibodies VRC01, B12, CH58, and CH22 was performed to yield 12 dilutions (range 1000–0.488 μg/ml). Diluted antibodies were added to the plates incubated for 1 hour, followed by 1-hour incubation with an HRP-conjugated goat anti-human IgG (Jackson ImmunoResearch, Pennsylvania). VRC01 and B12 target the CD4 binding site on the HIV envelope, whereas CH58 targets the V1V2 construct and CH22 targets the V3 construct. The plates were developed by using the SureBlue TMB microwell peroxidase substrate (SeraCare, Milford, MA) and the reaction was terminated with an equal volume of TMB stop solution (Sera care, Milford, MA). Optical density (OD) values at 450nm were measured with a SpectraMax Plus 384 plate reader (Molecular Devices, LLC.).

### Biolayer interferometry

Kinetics measurements of unconjugated 1086.C gp120 and gp120-Q11 nanofiber conjugates were performed on the OctetRED96 BLI platform using an orbital flow rate of 500 rpm (ForteBio, New York). Anti-hIgG Fc Capture (AHC) biosensors were equilibrated in phosphate-buffered saline (PBS; 1.06 mM potassium phosphate monobasic, 2.97 mM sodium phosphate dibasic, 155 mM sodium chloride) (60 s), loaded with CH65, VRC01, B12, CH58, or CH22 antibodies by submersion in 10 µg/mL solutions (300 s), and washed in PBS (60 s). An initial baseline was established in PBS (120 s) followed by an association phase in 0, 12.5, 25, 50, 100, 150, 200, 250 µg/mL 1086.C gp120 or gp120-Q11 nanofiber conjugate (400 s) and a dissociation phase in PBS (600 s). Kinetic analysis was performed using the ForteBio Data Analysis 10.0 software. Experimental curves were double-reference-subtracted using PBS buffer blanks and the CH65 reference sensors. The resultant curves were fit to a 2:1 (heterogeneous ligand) binding model and the reported dissociation rate constants (k_off_) represent the fast components of the model fit.

### Animal immunizations

Female 8-week-old, C57BL/6 mice were purchased from Jackson Laboratories, shipped to Duke University, and acclimated for one week prior to all immunization studies. Sample sizes were determined based on data from prior experiments with nanofiber immunogens. For primary evaluation of 1086c nanofibers, mice were subcutaneously immunized with 50 μg of gp120 (n=5), gp120-Q11 conjugates (n=4), or gp120/Q11 mixtures (n=5) followed by 3 boosts. To assess the immunogenicity of the vaccines in the presence an adjuvant, 3 doses of 15 μg gp120 or gp120-Q11 were used to immunize five mice per group in 10% of STR8S-C (v/v). Three groups of mice (n=5) in the multivalency experiment were immunized with 3 doses of 15 μg of gp120^high^-Q11, gp120^low^-Q11, or gp120-CP_3_Q11. Blood from each mouse was collected bi-weekly to measure the antibody responses. Researchers were not blinded to treatment groups when administering immunizations and collecting blood. All procedures were approved by the Duke University Institutional Animal Care and Use Committee under protocol A264-18-11, and reporting is in accordance with ARRIVE guidelines^61^. The study was carried out following all the relevant guidelines.

### ELISA analysis of antibody titers and avidity

Wells on 384-well polystyrene high-binding plates were coated with 1086.C gp120 (20 μg/mL) and blocked with superblock solution (4% whey, 15% goat serum, 0.5% Tween 20 in 1xPBS) for 1 h. Mouse sera were diluted in a tenfold series with superblock to yield six dilutions from 1:10 to 1:10^6^. The coated plates were incubated with the diluted serum samples at room temperature for 1 hour, then were washed 4 times with superwash buffer (0.1% Tween 20 in 1xPBS). HRP-conjugated goat anti-mouse antibody (Promega) in 1:5000 dilution was incubated in the same condition, followed by 4 washes. SureBlue TMB microwell peroxidase substrate was used for color development, with 5 min of incubation applied before the reaction was terminated with an equal volume of TMB stop solution. OD values at 450 nm was read with a SpectraMax Plus 384 plate reader. Serum antibody avidity was measured as previously described^62^. Plates were coated and treated with serum as described above, then treated with urea solution (7M in water) for 5 min, followed by 4 washes and an 1-hour incubation of detection antibody. Avidity indices were calculated according to the following formula: (mean OD in urea-treated wells/mean OD in PBS-treated wells) × 100.

### Binding antibody multiplexed assay (BAMA)

HIV-1 specific antibodies were measured using a custom binding antibody multiplex assay similar to those previously described^62–64^. Carboxylated fluorescent beads (Luminex Corp) were covalently coupled to a panel of five house-made HIV-1 antigens: 1086C. gp120, MN. gp120 gDneg (clade B), A244 gp120 gDneg (Clade AE), A1.con.env03 gp140 (consensus clade A envelope gp140 with deletions in the gp41 cleavage site and fusion domain), and B.con.env03 gp140 (consensus clade B envelope gp140). Mouse sera were diluted in diluent (1% skim milk, 5% normal goat serum, 0.05% tween 20 in 1X PBS; pH 7.4) to generate six 6-fold serially diluted samples starting from 1:10 dilution. The diluted samples were incubated with coated beads against each antigen (bead count > 3500 for each antigen) in Millipore Sigma 1.2 μm 96-well filter plates at room temperature for 30 min, followed by 3 washes with wash buffer (0.1% BSA, 0.02% Tween 20, 0.05% sodium azide in 1X PBS; pH 7.4) and vacuum manifold was used to remove the wash buffer while the filter plate retained the beads in the wells. PE-conjugated goat anti-Mouse IgG (SouthernBiotech) (2 μg/ml) was dispensed to each well and the plates were incubated at room temperature for 30 min. After washing 3 times with wash buffer, the plates were read by a Bio-Plex 200 System. Results were expresses as mean fluorescence intensity (MFI). In this assay, serum pooled from 13 gp120-immunized mice was used as the positive control, and pre-immunized serum pooled from the same group of mice was used as the negative control.

### Fluorescent labeling of antigens for flow cytometry

To detect gp120-specific B cells by flow cytometry, we synthesized fluorescently labeled 1086.C gp120 for cell staining. Avi-tagged 1086.C gp120 was biotinylated using BirA biotin-protein ligase standard reaction kit (Avidity, BirA500) following the manufacturer’s protocol. Tetramers were prepared based on the molar ratio (4:1) of the analyte protein and fluorochrome-conjugated streptavidin, respectively. Alexa Fluor 647 (ThermoFisher, S21374) or Brilliant Violet 421 (BioLegend, 405225) conjugated streptavidin was reacted with the biotinylated protein over 5 additions, incubating for 15 min between each addition. The final concentration of tetramer was calculated with respect to the analyte protein after which PBS was added to achieve concentration of 3 µM. The solution was aliquoted, snap frozen, and stored at −80°C.

### Flow cytometry of lymph node-isolated cells

Harvested mouse inguinal, brachial, and axillary nodes were harvested by unblinded researchers and gently ground against a 70 μm Falcon cell strainer (VWR international LLC.) to break down the tissue, followed by purification using Lympholyte-M Cell Separation Media (Cedarlane). Lymphocyte density and viability were measured by MUSE Cell Analyzer (Sigma-Millipore). For B cell phenotyping, 2x10^6^ total cells were stained with fluorescently labeled antibodies IgG1 FITC, IgG2 FITC, IgG3 FITC, CD93 PE-CF954, IgM PE-Cy7, CD19 APC-R700, CD95 BV605, B220 BV650, CD138 BV711, CD23 BV786, and IgD BV510 (all from BD Biosciences), GL7 PE, CD21 PerCP-Cy5.5, CD11b BV570 (both from BioLegend), CD38 PE-Cy5 (eBioscience). For T cell phenotyping, 1x10^6^ lymphocytes were stained with a panel of antibodies including CD4 FITC, CD25-PE, CD279 PE-CF594, CD62L PE-Cy7, CXCR5 Biotin, CD8a APC-R700, CD127 BV421, CD3e BV510, CD90.2 BV605, CD44 BV711, and B220 BV786 (all from BD Biosciences), NK1.1 BV650, CD11b BV570, and CD49b PerCP-Cy5.5, and TER119 PE-Cy5 (all from Biolegend), followed by incubation with Streptavidin AF647 (Invitrogen) for CXCR5 staining. After washing with PBS, cells stained with the B-cell or T-cell panel were incubated with Live/Dead near-IR dye (Invitrogen) in 1:1000 dilution. Cells were then fixed with 2% formaldehyde in PBS and analyzed using a BD LSRII flow cytometer (BD Biosciences). Analysis of specific cell populations was executed following the gating schemes shown in Figs. S11 and S12.

#### Antigen tracking with fluorescence molecular tomography (FMT)

To measure the drainage of immunogens from the subcutaneous space after injection, gp120 conjugates were prepared as described above and then labeled with VivoTag647 (Perkin Elmer). After conjugation, gp120^high^-Q11 and gp120^low^-Q11 fibers were centrifuged and washed with PBS to remove unconjugated fluorophores. Similarly, gp120-CP_3_Q11 conjugates were dialyzed in PBS to remove unconjugated fluorophores. Labeled immunogens were then diluted in PBS to equal gp120 concentration and 15 μg of gp120 was injected subcutaneously on the right and left flanks of SKH-1 mice. A control mouse which received no injection was also imaged and used to set thresholds for background fluorescence intensity. Fluorescence was measured over 6 days using a Perkin Elmer VisEn FMT2500LX and fluorescenece intensity was quantified in TrueQuant software.

#### Statistical analysis

Statistical analyses involving serum ELISA data were completed using linear mixed effects, with fixed effects for vaccine, booster period, and week along with nested random effects for individual mice by booster period. All analyses were done within R using the lme4^65^ and lmerTest^66^ packages using an ANOVA for linear effects models. Statistical analyses for BAMA data were done using Generalized Estimating Equations (GEE)^67^. We evaluated responses across all time points with differing effects for antigen and assessed the interaction between vaccine and week at which the data was collected. Due to the small sample size, we used bias-corrected standard error estimators^68^. Testing was done using Wald test-statistics. Multiple testing correction was done via the Holm procedure^69^, with the significance threshold post-correction at 0.05. In addition, we used a non-parametric repeated measures test^70^ when noted in figure legends. No treated animals were excluded from statistical analysis.

## Supplementary Information

Supplementary Information.
