# Supplementary material for: HIV envelope antigen valency on peptide nanofibers modulates antibody magnitude and binding breadth
Source: Sci Rep. 2021 Jul 14;11:14494. doi: 10.1038/s41598-021-93702-x (PMC8280189; doi:10.1038/s41598-021-93702-x)
Supplement: Supplementary file 1 — Supplementary Information. [file 41598_2021_93702_MOESM1_ESM.docx]

***Supplementary Information***

**HIV envelope antigen valency on peptide nanofibers modulates antibody magnitude and binding breadth**

# Chelsea N. Fries,^1,+^ Jui-Lin Chen,^2,3+^ Maria Dennis,^3^ Nicole L. Votaw,^1^ Joshua Eudailey,^3^ Brian E. Watts,^3^ Kelly M. Hainline,^1^ Derek W. Cain,^3^ Richard Barfield,^4^ Cliburn Chan,^4^ M. Anthony Moody,^3,5,6^ Barton F. Haynes,^3,6^ Kevin O. Saunders,^2,3,6,7^ Sallie R. Permar,^2,3,5,6,8^ Genevieve G. Fouda,^2,3,5^* Joel H. Collier^1,6^*­

^+^these authors contributed equally to this work

^1^ Dept of Biomedical Engineering, Duke University, Durham, NC 27708

^2^Department of Molecular Genetics and Microbiology, Duke University School of Medicine, Durham, NC 27710

^3^Duke Human Vaccine Institute, Duke University School of Medicine, Durham NC 27710

^4^Department of Biostatistics and Bioinformatics, Duke University School of Medicine, Durham NC 27710

^5^Department of Pediatrics, Duke University Medical Center, Durham, NC 27710

^6^Department of Immunology, Duke University School of Medicine, Durham, NC 27710

^7^Department of Surgery, Duke University School of Medicine, Durham, NC 27710

^8^Department of Pediatrics, New York-Presbyterian/Weill Cornell Medicine, New York, NY 10065

**Key Reagents**

| Reagent | Source | Identifier |
| --- | --- | --- |
| Antibody |  |  |
| HRP-conjugated goat anti-human IgG | Jackson ImmunoResearch | Cat# 109-035-008 |
| HRP-conjugated goat anti-mouse IgG | Promega | Cat# W402B |
| PE-conjugated goat anti-mouse IgG | SouthernBiotech | Cat# 1030-09S |
| Human anti-HIV VRC01 IgG | Zhou et al. 2010^45^ | N/A |
| Human anti-HIV B12 IgG | Zwick et al. 2003^46^ | N/A |
| Human anti-HIV CH58 IgG | Liao et al. 2013^47^ | N/A |
| Human anti-HIV CH22 IgG | Santra et al. 2015^48^ | N/A |
| Human anti-HIV CH65 IgG | Whittle et al. 2011^49^ | N/A |
| Rat anti-mouse IgG1 FITC | BD Biosciences | Cat# 553443 |
| Rat anti-mouse IgG2a/2b FITC | BD Biosciences | Cat# 553399 |
| Rat anti-mouse IgG3 FITC | BD Biosciences | Cat# 553403 |
| Rat anti-mouse/human GL7 PE | BioLegend | Cat# 144608 |
| Rat anti-mouse CD93 PE-CF954 | BD Biosciences | Cat# 563805 |
| Rat anti-mouse IgM PE-Cy7 | BD Biosciences | Cat# 552867 |
| Rat anti-mouse CD19 APC-R700 | BD Biosciences | Cat# 565473 |
| Hamster anti-mouse CD95 BV605 | BD Biosciences | Cat# 740367 |
| Rat anti-mouse B220 BV650 | BD Biosciences | Cat# 563893 |
| Rat anti-mouse CD138 BV711 | BD Biosciences | Cat# 563193 |
| Rat anti-mouse CD23 BV786 | BD Biosciences | Cat# 563988 |
| Rat anti-mouse IgD BV510 | BD Biosciences | Cat# 563110 |
| Rat anti-mouse CD21 PerCP-Cy5.5 | BioLegend | Cat# 123416 |
| Rat anti-mouse CD11b BV570 | BioLegend | Cat# 101233 |
| Rat anti-mouse CD38 PE-Cy5 | eBioscience (Invitrogen) | Cat# 15-0381-82 |
| Rat anti-mouse CD4 FITC | BD Biosciences | Cat# 553047 |
| Rat anti-mouse CD25 PE | BD Biosciences | Cat# 558642 |
| Hamster anti-mouse CD279 PE-CF594 | BD Biosciences | Cat# 562523 |
| Rat anti-mouse CD62L PE-Cy7 | BD Biosciences | Cat# 560516 |
| Rat anti-mouse CXCR5 Biotin | BD Biosciences | Cat# 551960 |
| Rat anti-mouse CD8a APC-R700 | BD Biosciences | Cat# 564983 |
| Rat anti-mouse CD127 BV421 | BD Biosciences | Cat# 562959 |
| Hamster anti-mouse CD3e BV510 | BD Biosciences | Cat# 563024 |
| Rat anti-mouse CD90.2 BV605 | BD Biosciences | Cat# 105343 |
| Rat anti-mouse CD44 BV711 | BD Biosciences | Cat# 563971 |
| Rat anti-mouse B220 BV786 | BD Biosciences | Cat# 563894 |
| Rat anti-mouse NK1.1 BV650 | BioLegend | Cat# 108736 |
| Rat anti-mouse CD49b PerCP-Cy5.5 | BioLegend | Cat# 108916 |
| Rat anti-mouse TER119 PE-Cy5 | BioLegend | Cat# 116210 |
| Chemicals, peptides and recombinant proteins | | |
| Q11 | Rudra et al. 2010^50^ | N/A, sequence in Table S1 |
| C-Q11 | Derivative of Rudra et al. 2010^50^ | N/A, sequence in Table S1 |
| C-P_3_Q11 | Rudra et al. 2012^26^ | N/A, sequence in Table S1 |
| sulfosuccinimidyl 4-(N-maleimidomethyl) cyclohexane-1-carboxylate (Sulfo-SMCC) | ThermoFisher | Cat# 22322 |
| Streptavidin AF647 conjugate | Invitrogen | Cat# S32357 |
| LIVE/DEAD Fixable Near-IR Dead Cell Stain | Invitrogen | Cat# L10119 |
| 1086.C gp120 | Go et al. 2013 | N/A |
| MN. gp120 gDneg | Rerks-Ngarm et al. 2009^51^ | N/A |
| A244 gp120 gDneg | Rerks-Ngarm et al. 2009^51^ | N/A |
| A1.con.env03 gp140 | Haynes et al. 2012^52^ | N/A |
| B.con.env03 gp140 | Rerks-Ngarm et al. 2009^51^ | N/A |
| Commercial Assay Kit | | |
| Coating Solution Concentrate Kit | SeraCare | Cat# 5150-0014 |
| TMB microwell peroxidase substrate | SeraCare | Cat# 5120-0083 |
| TMB stop solution | SeraCare | Cat# 5150-0021 |
| Other | | |
| 384-well polystyrene high-binding plates | Corning | Cat#3700 |
| Formvar-coated copper TEM grids | Electron Microscopy Sciences | Cat#FCF400-Cu-SC |
| MilliporeSigma 1.2 μm 96-well filter plates | Millipore | Cat# MSBVN1250 |
| Anti-hIgG Capture Tips | ForteBio | Cat #18-5060 |

**Table S1.** Peptide Sequences

| Q11 | Ac-QQKFQFQFEQQ-NH_2_ |
| --- | --- |
| C-Q11 | Ac-C-SGSG-QQKFQFQFEQQ-NH_2_ |
| CP_3_Q11 | Ac-C-SGSG-QQKPQPQPEQQ-NH_2_ |

Ac: acetylated N-terminus; NH_2_: amidated C-terminus

**Table S2.** Estimated antigens per nanofiber from mouse immunizations. Antigens per fiber were estimated based on the concentration of gp120 in each sample, the crystal structure of a β-sheet amyloid (PDB 2nnt), and the average length of measured nanofibers.


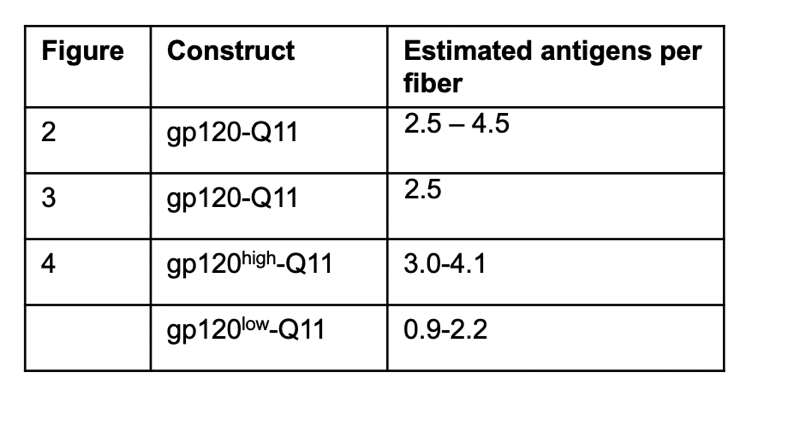

**Figure S1.** Binding of gp120-specific monoclonal antibodies to CQ11 nanofibers by ELISA. These measurements were taken for nanofibers adsorbed directly to ELISA plates, then treated with mAbs listed in the legend, followed by a detection antibody. No detectable background binding was observed.


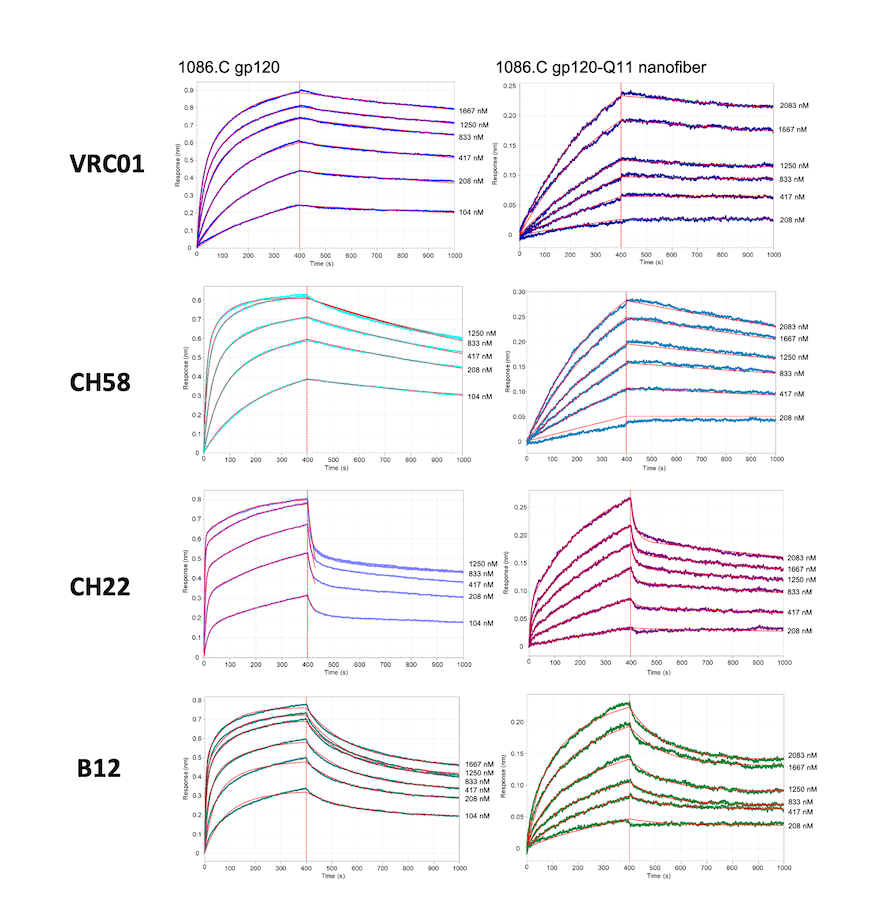


**Figure S2.** Biolayer Interferometry (BLI) binding of mAbs to gp120-Q11. Full experimental details for BLI are listed in the methods. Briefly, anti-human capture tips were dipped in wells with the mAbs of interest (left hand text), followed by submersion in wells with varying concentrations of gp120 or gp120-Q11. Association and dissociation rate constants (k_on_ and k_off_) were measured by detecting binding events in gp120 wells, followed by dissociation of the constructs when tips are transferred to wells containing PBS.


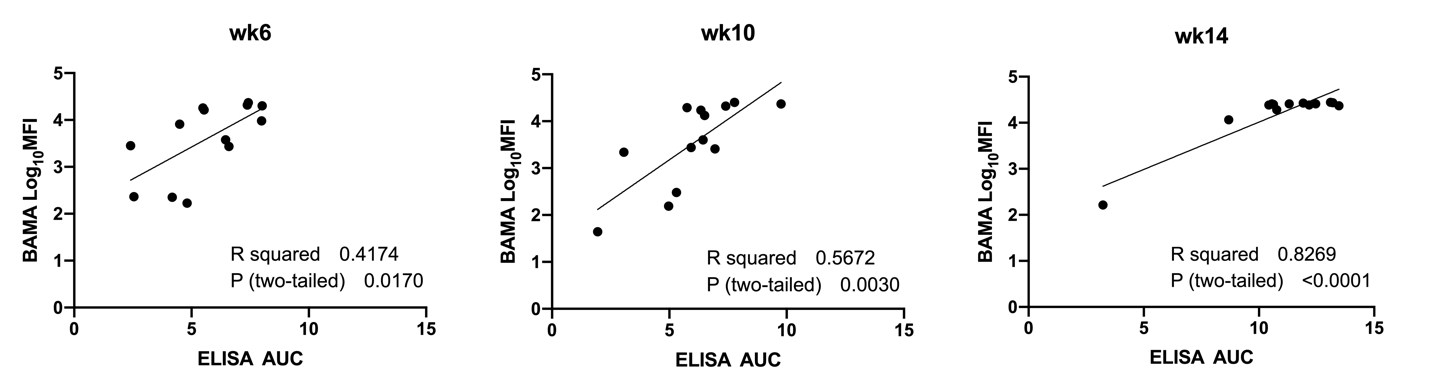


**Figure S3.** Correlation between data generated with ELISA and BAMA assays for antibody responses against 1086c. Each point represents the serum sample from each animal in Figure 2.


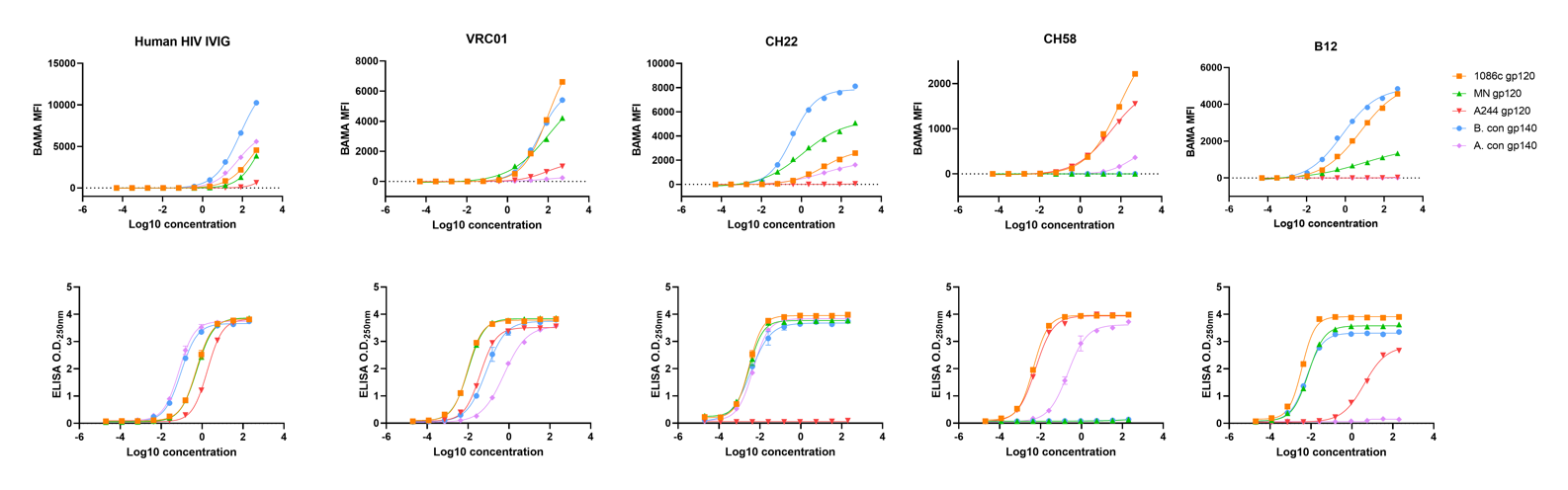


**Figure S4.** Binding curves of polyclonal anti-HIV IVIG and mAbs to BAMA panel antigens in BAMA (top row) and ELISA (bottom row).


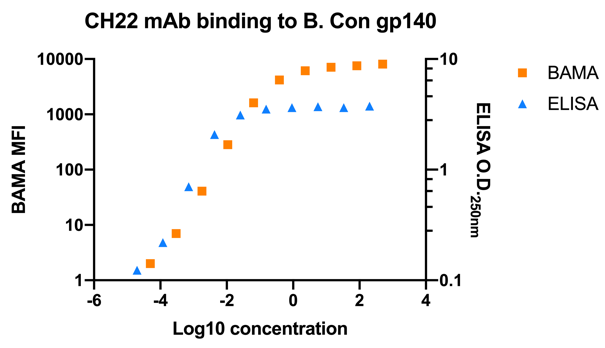


**Figure S5.** The dynamic range of BAMA and ELISA is illustrated with the CH22 mAb/ Con gp140 antibody-antigen pair, both of which exhibit sigmoidal binding curves.

**
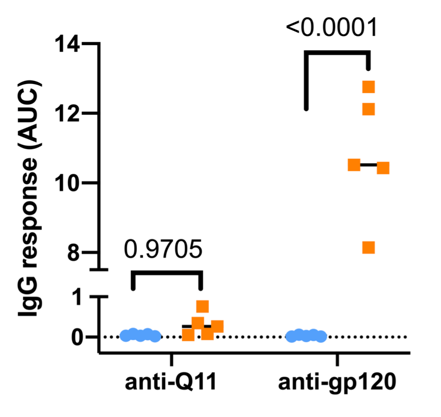
**

**Figure S6**. Anti-Q11 and anti-gp120 IgG antibodies measured from the serum of mice immunized with gp120-Q11 + STR8SC (Figure 3). Groups were compared by two-way ANOVA with Tukey’s post hoc comparison.

**
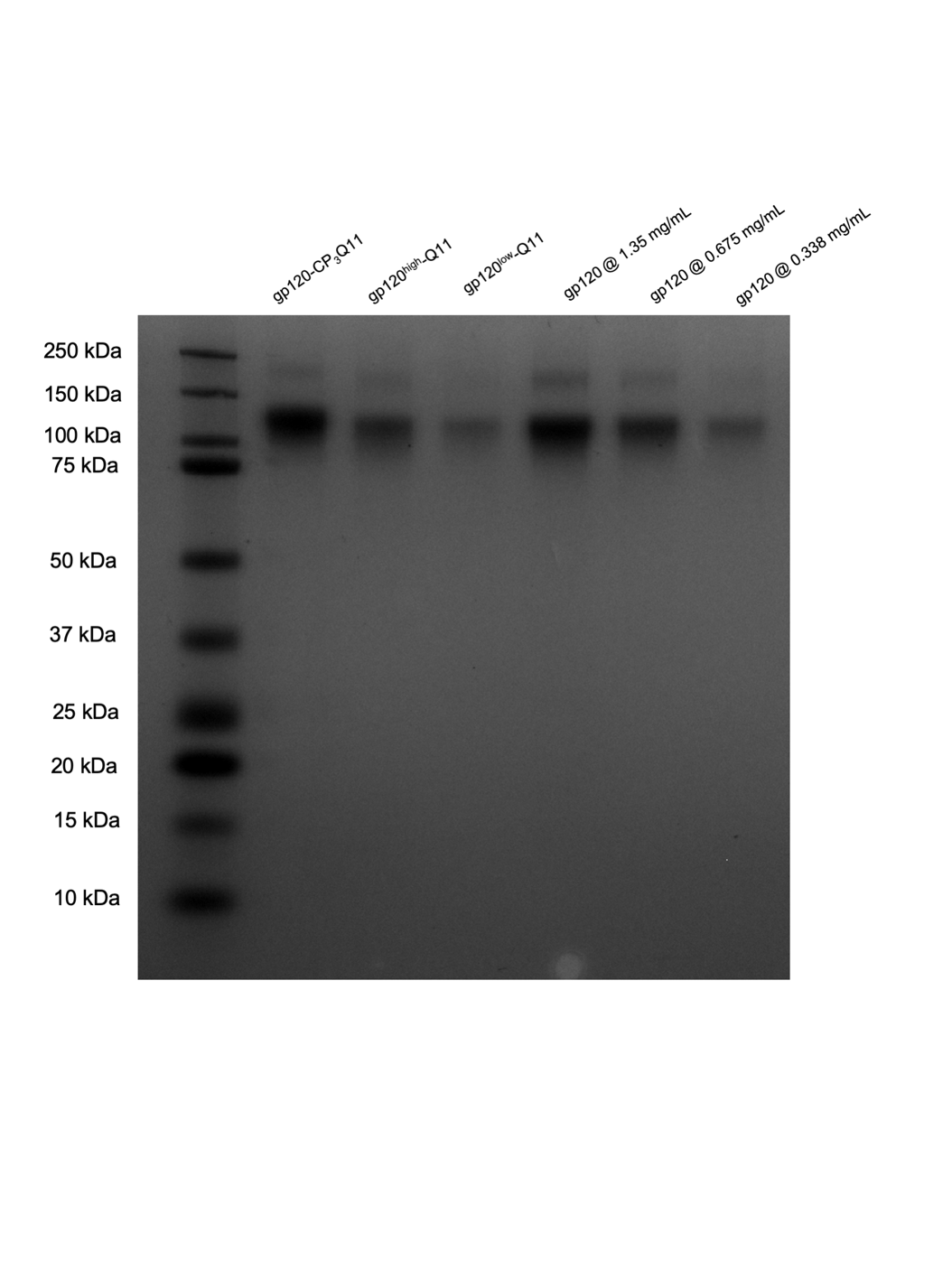
**

**Figure S7.** Characterization of gp120 content on high- and low-loading gp120-Q11 vaccines. Intensity of SDS-PAGE bands was used to determine gp120 content in vaccine constructs (lanes 2-4) using a standard curve created by gp120 standards (lanes 5-7). High- and low-loading gp120-Q11 have equal concentrations of nanofiber-forming Q11 peptides, and their relative intensities illustrates differences in gp120 density in the two constructs.


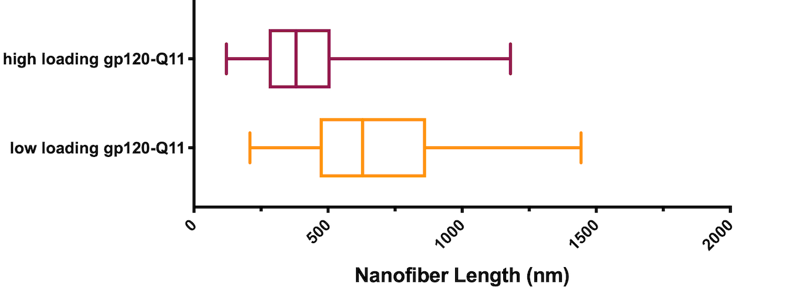


**Figure S8.** Length distributions of gp120^high^-Q11 and gp120^low^-Q11 nanofibers. TEM images of high- and low-loading gp120-Q11 were traced in ImageJ and used to calculate the length ranges of nanofibers. Plot whiskers show minimum and maximum fiber lengths, and boxes show 25^th^, 50^th^, and 75^th^ percentiles. Lengths were compared by a Wilcoxon Rank sum test with continuity correction and were significantly different (p < 5*10^-15^).

**
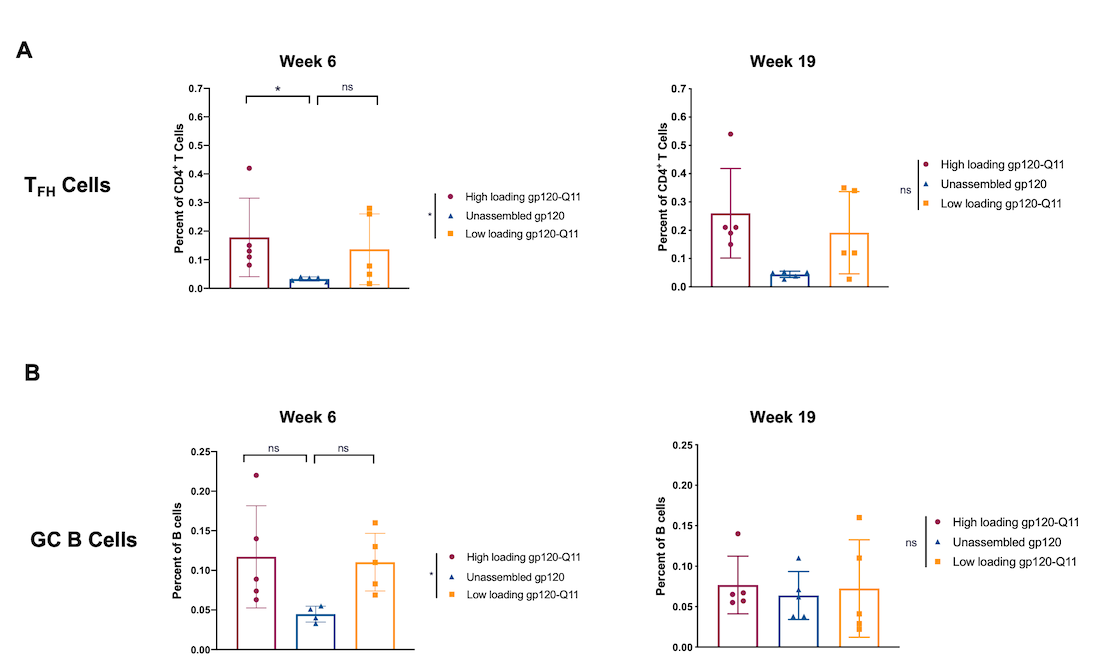
**

**Figure S9.** Percentage of antigen-specific germinal center B cells and T_FH_ cells after immunization with high- and low-loading gp120-Q11 nanofibers. **A)** Percent of CD4^+^ T cells with a T_FH_ phenotype (CD3^+^ CD4^+^ CD44^hi^ CD62L^Lo^ CD25^-^ CD279^hi^ CXCR5^hi^) at 6 -and 19-weeks post-priming immunization. **B)** Percent of B Cells with a GC B cell phenotype and double positive binding to 1086.C gp120 (CD138^-^ B220^+^ GL7^hi^ ) at 6 -and 19-weeks post-priming immunization. Ranks were compared using the Kruskal-Wallis test (n=5 animals/group). Differences in cell percentage due to treatment group are shown next to figure legends and differences between specific groups tested by post-hoc pairwise comparisons are shown above the data.

**
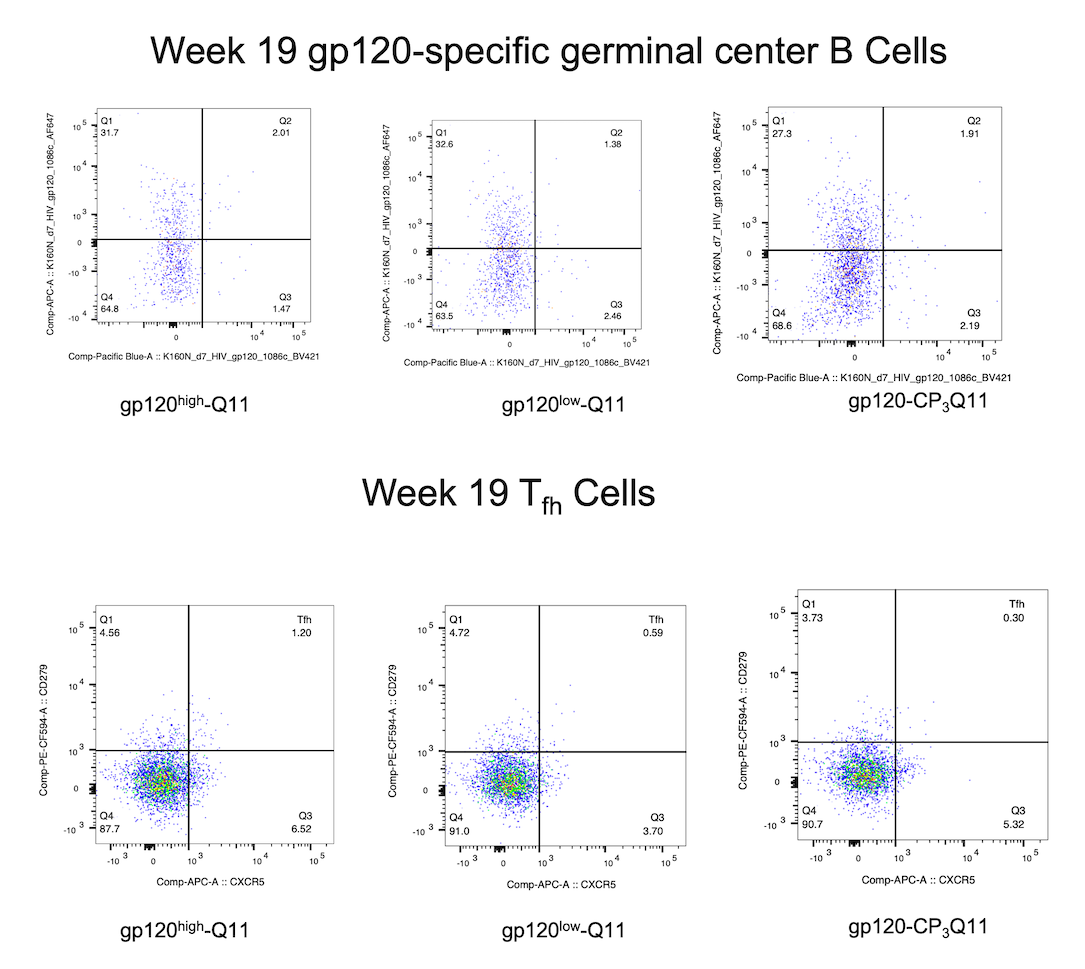
**

**Figure S10.** Representative dot plots of T_FH_ and gp120-specific germinal center B cells isolated from lymph nodes of mice immunized with gp120-Q11 at week 19.


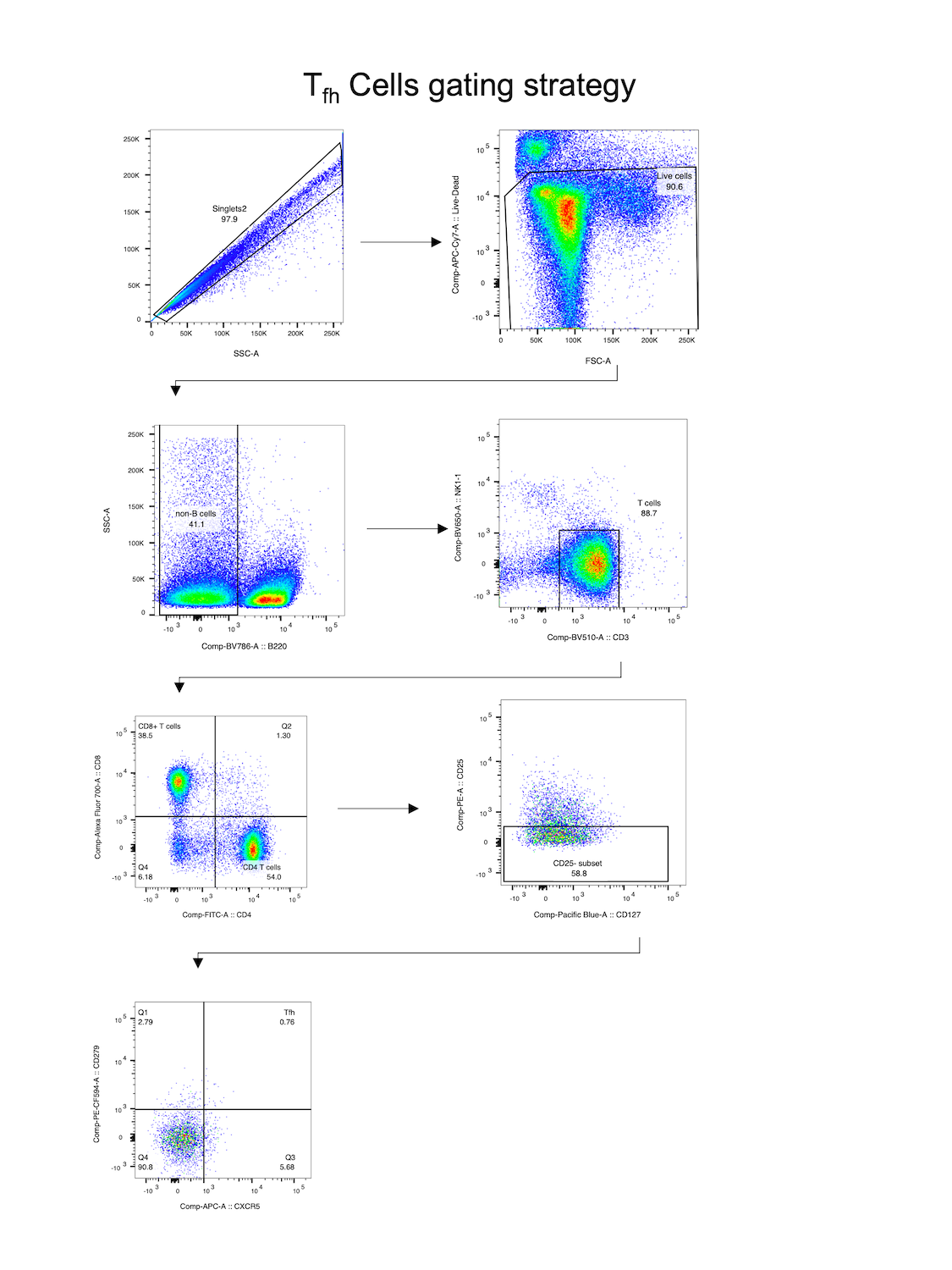


**Figure S11.** Flow cytometry gating scheme for detection of T_FH_ cells.


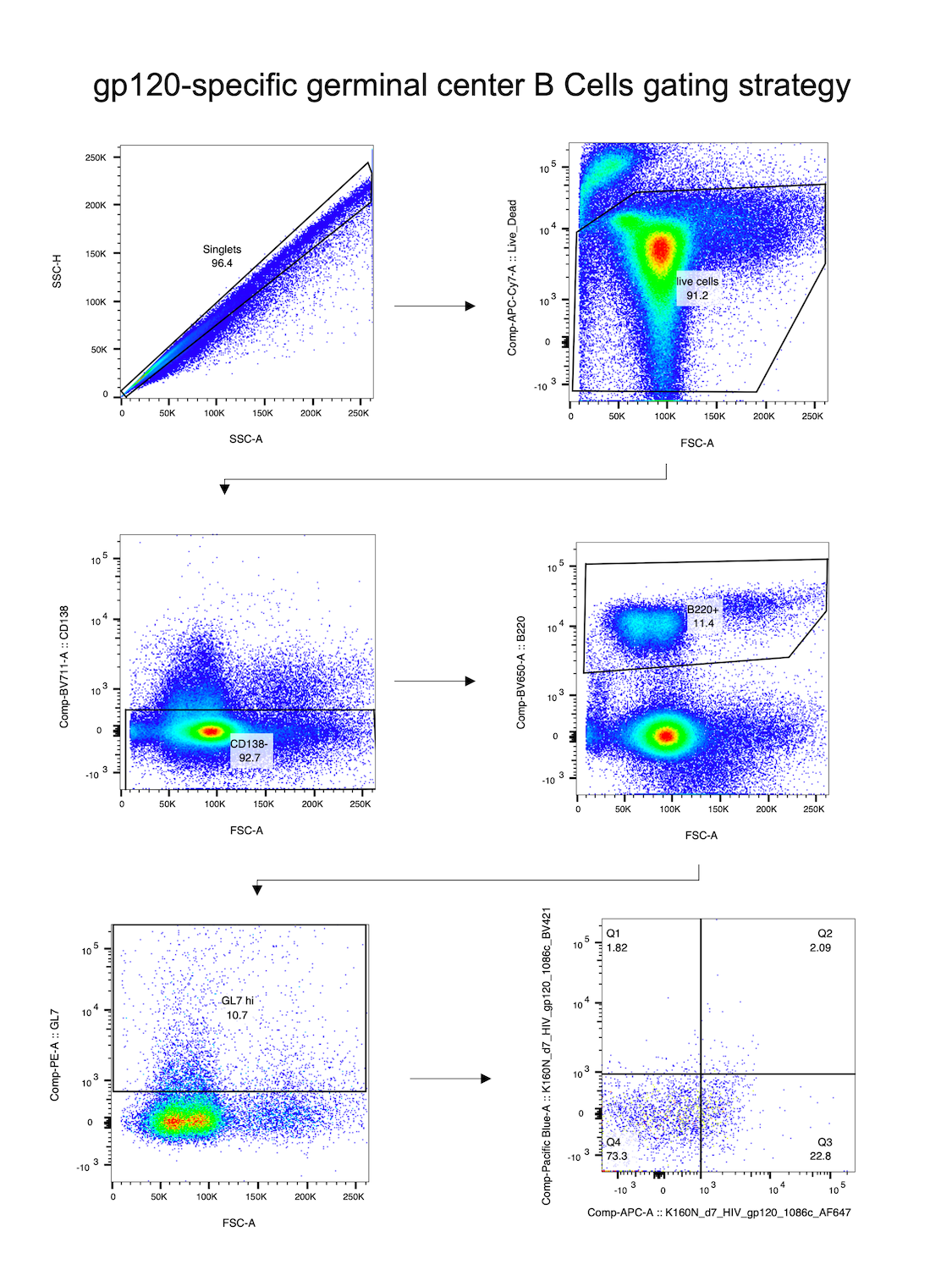


**Figure S12.** Flow cytometry gating scheme for detection of gp120-specific B cells.

**Figure S13 (below).** FMT images of mice immunized with fluorescently labeled gp120 immunogens. At each time point, mice injected with fluorescently labeled gp120 vaccines (panels 2-4) were compared to a control mouse with no injection (panel 1). Images are displayed with all the time points of a single treatment group in one page.


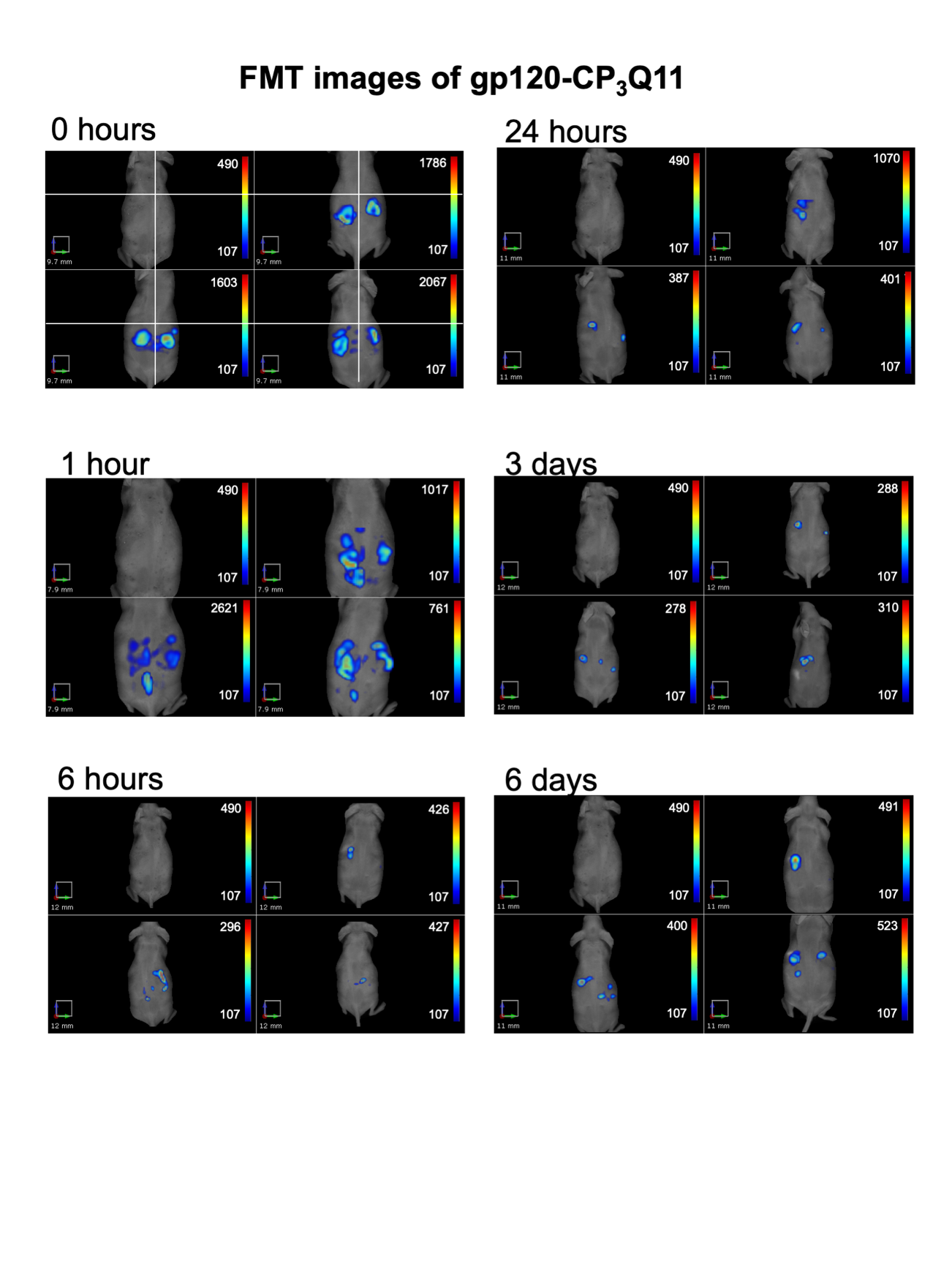


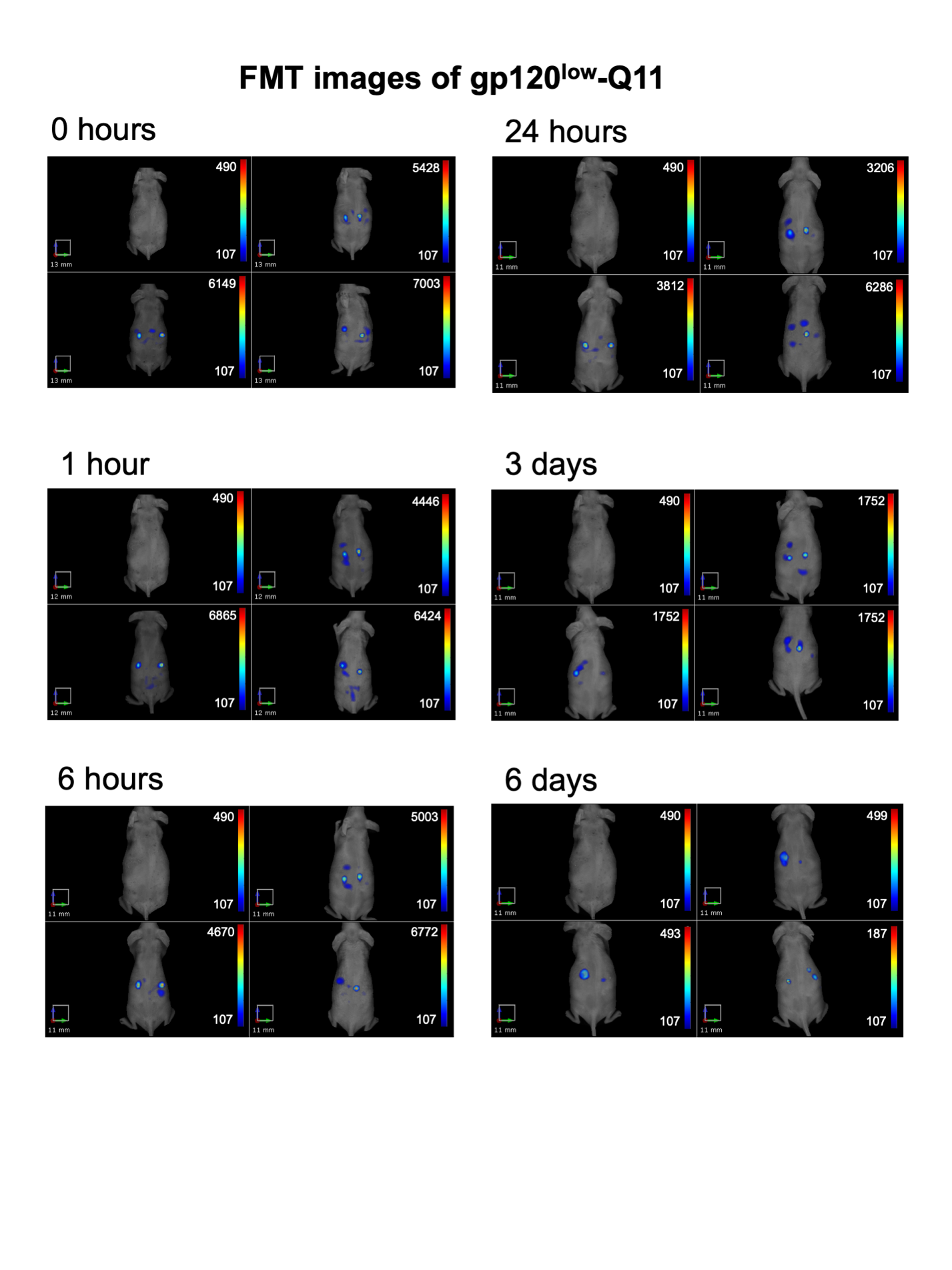


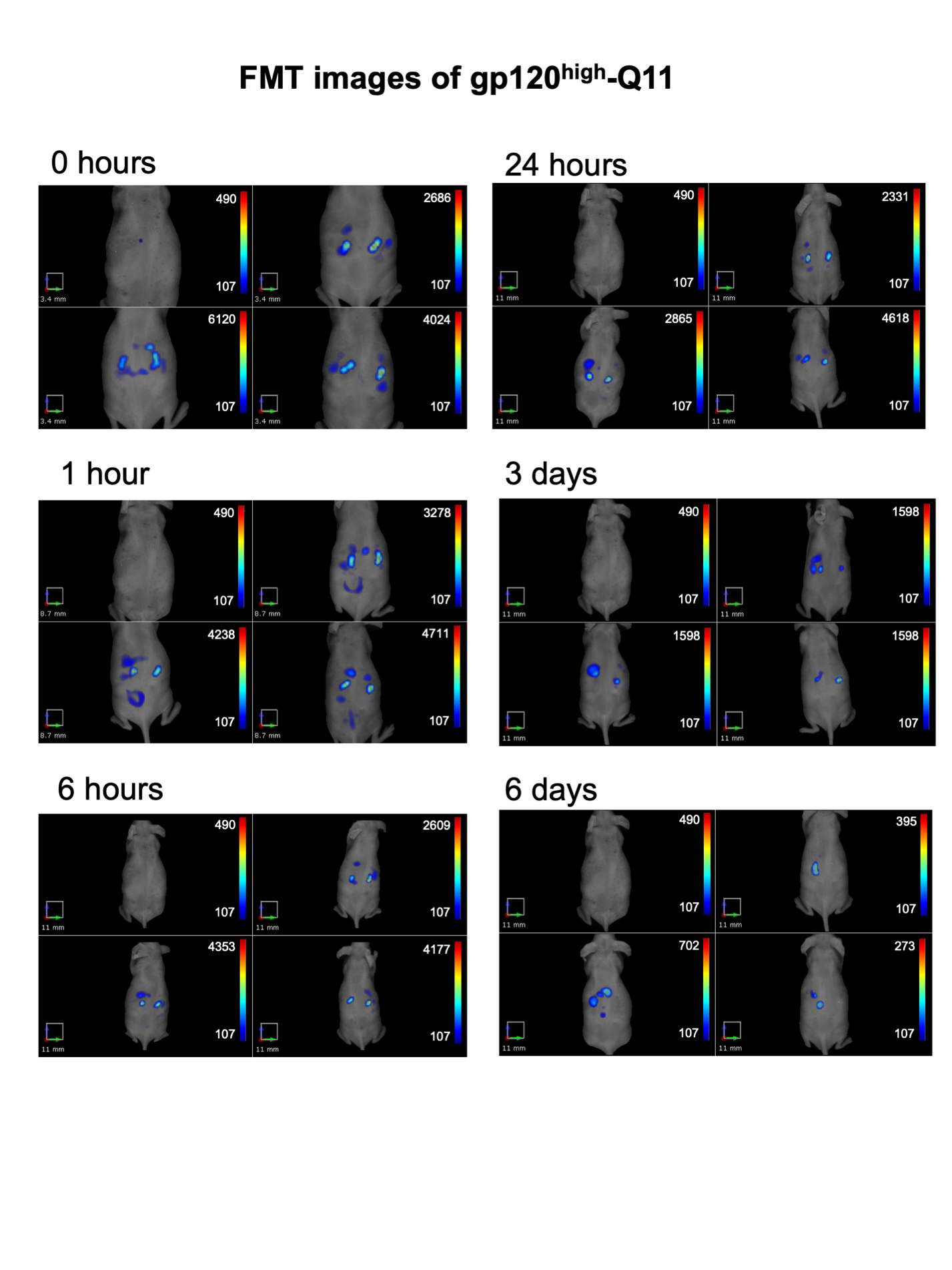


**Figure S14 (below).** Angled FMT images of mice from Figure 6 and Figure S13 to show proximity of fluorescence to skin surface.


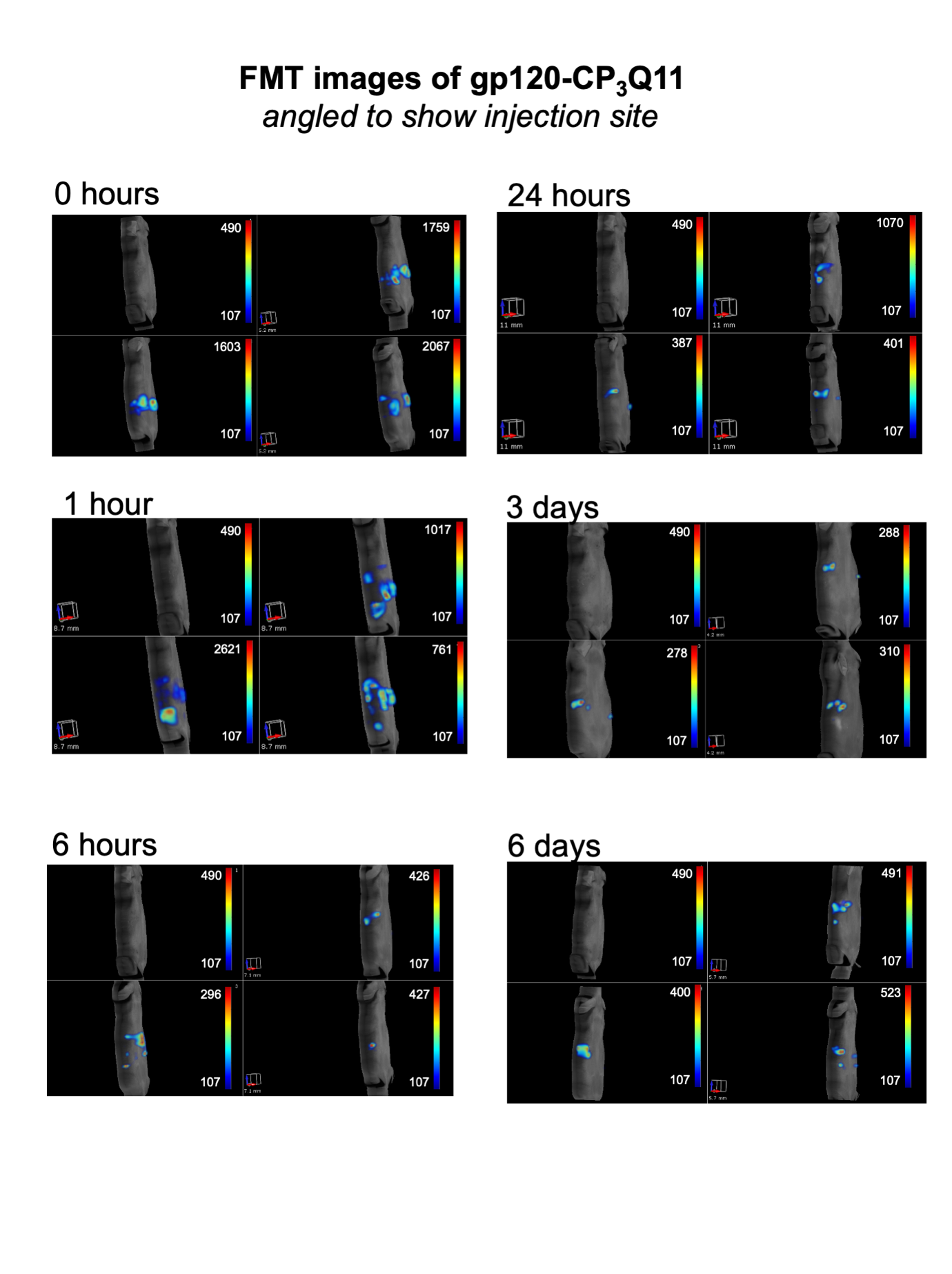


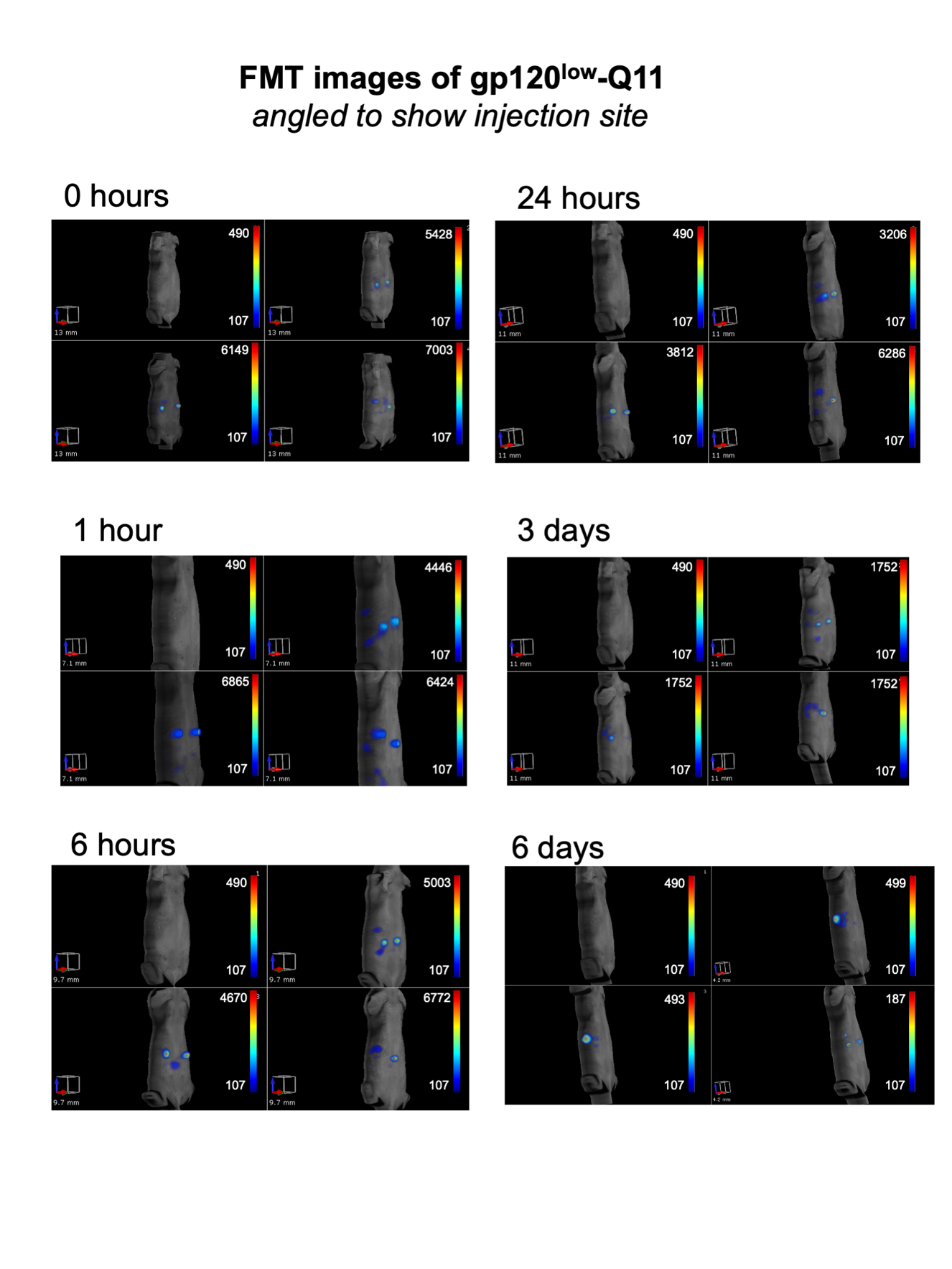


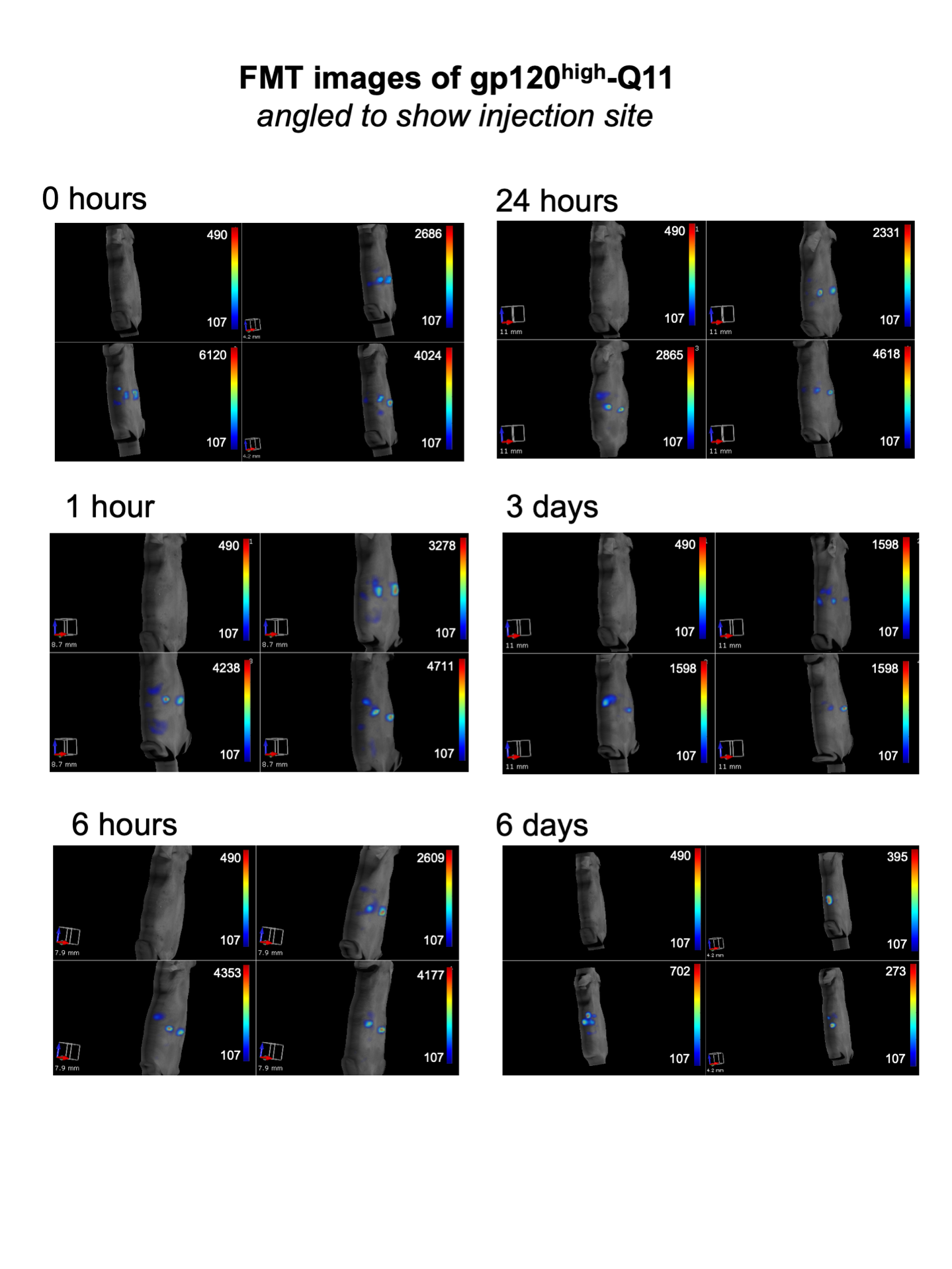

**Figure S15.** Raw values of fluorescent material detected in excised skin of mice from Figure 6.
